# The functional role of sequentially neuromodulated synaptic plasticity in behavioural learning

**DOI:** 10.1371/journal.pcbi.1009017

**Published:** 2021-06-10

**Authors:** Grace Wan Yu Ang, Clara S. Tang, Y. Audrey Hay, Sara Zannone, Ole Paulsen, Claudia Clopath

**Affiliations:** 1 Department of Bioengineering, Imperial College London, South Kensington Campus, London, United Kingdom; 2 Department of Physiology, Development and Neuroscience, Physiological Laboratory, Cambridge, United Kingdom; Dartmouth College, UNITED STATES

## Abstract

To survive, animals have to quickly modify their behaviour when the reward changes. The internal representations responsible for this are updated through synaptic weight changes, mediated by certain neuromodulators conveying feedback from the environment. In previous experiments, we discovered a form of hippocampal Spike-Timing-Dependent-Plasticity (STDP) that is sequentially modulated by acetylcholine and dopamine. Acetylcholine facilitates synaptic depression, while dopamine retroactively converts the depression into potentiation. When these experimental findings were implemented as a learning rule in a computational model, our simulations showed that cholinergic-facilitated depression is important for reversal learning. In the present study, we tested the model’s prediction by optogenetically inactivating cholinergic neurons in mice during a hippocampus-dependent spatial learning task with changing rewards. We found that reversal learning, but not initial place learning, was impaired, verifying our computational prediction that acetylcholine-modulated plasticity promotes the unlearning of old reward locations. Further, differences in neuromodulator concentrations in the model captured mouse-by-mouse performance variability in the optogenetic experiments. Our line of work sheds light on how neuromodulators enable the learning of new contingencies.

## Author summary

Reversal learning likely involves changes in synaptic connections, a neural mechanism known as synaptic plasticity, so old information can be updated. We previously discovered that acetylcholine, an important neuromodulator in the brain, changes synaptic connections in a way that favours reversal learning. Specifically, acetylcholine weakens active synapses in brain slices, but these synapses can later be strengthened by a reward signal. Based on this result in slices, we used a computational model to propose a behavioural function for the action of acetylcholine on synaptic connections. In the model, acetylcholine would weaken synaptic connections associated with an old reward, allowing an agent to quickly learn a new reward location. We tested this hypothesis here by silencing acetylcholine neurons in mice while they navigated a maze for food rewards. These animals were able to learn the location of the first food reward, but were impaired when the reward was shifted to a new location. The behavioural results of this study suggest that acetylcholine indeed facilitates reversal learning, which the computational model attributes to a weakening of synaptic connections that do not lead to reward. Taken together, our experimental and computational work show how synaptic strength changes, gated by neuromodulators, affect learning behaviour.

## Introduction

When the environment changes and previous reward associations no longer hold, an animal must quickly adapt its behaviour to maximize reward. The learning rules in the brain responsible for updating action-outcome contingencies in such situations are not fully understood. Traditional forms of Hebbian plasticity [1, 2], including spike-timing-dependent-plasticity (STDP) [3–7], change synaptic weights based on the joint activation of pre- and post- synaptic neurons alone. They do not account for behavioural learning paradigms that require external feedback. Synaptic plasticity that is regulated by neuromodulators [8–11] provides a mechanism to incorporate behaviourally relevant information into synaptic changes, and at the appropriate time. Neuromodulatory signals are released in response to certain salient events (e.g. reward discovery or reward removal) and gate plasticity, depressing or potentiating recently active synapses responsible for the outcome, changing behaviour in a task relevant way [12].

Previous studies have examined either how neuromodulators regulate hippocampal plasticity [13–16] or how they affect behavioural functioning [17–19], but not together. Our work seeks to connect synaptic level changes to behaviour. Using experimental and computational means, we investigate the mechanisms through which neuromodulated-plasticity in the hippocampus influences reward learning. In our previous study, we uncovered in the hippocampus a form of neuromodulated synaptic plasticity that depends on the sequential modulation of two neuromodulators, acetylcholine (ACh) and dopamine (DA) [20]. The presence of acetylcholine produced synaptic depression during an STDP induction protocol in hippocampal slices. Adding dopamine after the induction protocol, within a time window of up to a minute, converted the acetylcholine-facilitated depression into potentiation. We termed this sequentially neuromodulated plasticity (sn-Plast), and formalized it as a learning rule [21]. Under the sn-Plast rule, a symmetric STDP window changes synaptic weights according to spike coincidences, irrespective of timing order, and the neuromodulator determines the sign of the weight change. Tonically-released acetylcholine depresses synapses, while a subsequent phasic dopamine signal retroactively converts depression into potentiation, through an eligibility trace that tracks active synapses. We hypothesized that this learning rule would be functionally important, since dopamine has been associated with reward expectation [22–25] and acetylcholine with exploration [26, 27], surprise and novelty [28–30]. To test the behavioural implications of our synaptic plasticity findings, we implemented sn-Plast in a spiking neural network model for reward-based navigation. Our simulations showed that sn-Plast agents unlearnt a previously rewarded location more quickly to find a new reward [20, 21]. This was because during exploration, cholinergic-facilitated depression weakened synapses and state-action associations that no longer led to the reward.

In this study, we performed the behavioural experiments to verify predictions from the sn-Plast model and previous slice experiments. Cholinergic neurons were optogenetically inactivated in mice during a hippocampus-dependent spatial navigation task assessing reversal learning. We show that the model captures the selective deficit in reversal learning caused by the optogenetic manipulation, and explains inter-individual variability in performance. These results further demonstrate that the sequential neuromodulation of STDP by acetylcholine and dopamine facilitates the learning of a new reward location.

## Results

### Silencing cholinergic neurons selectively impairs reversal learning but not initial place learning, as predicted by the sn-Plast model

**Fig 1:**
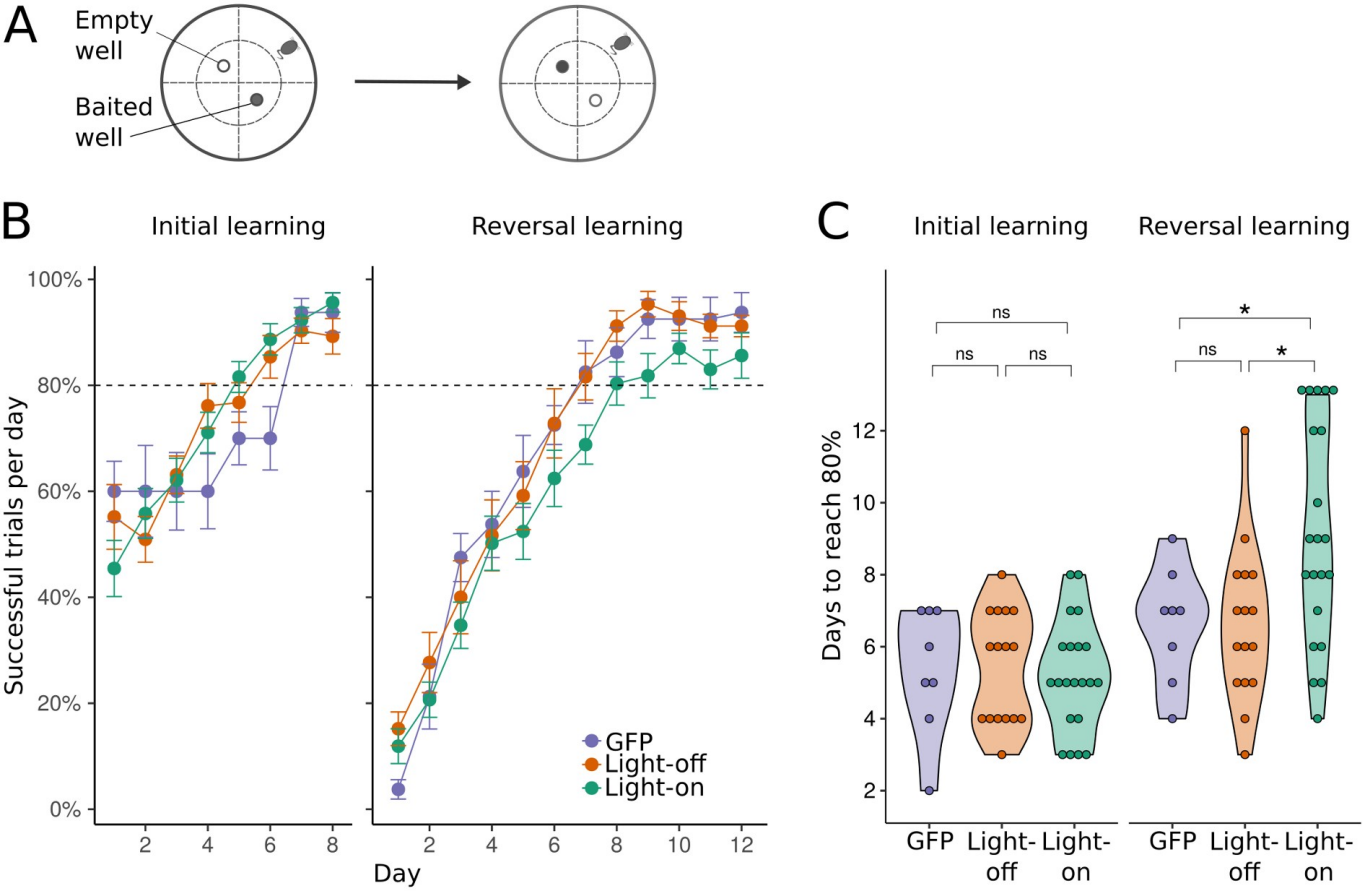
Inactivating cholinergic neurons affects reversal learning. (A) Schematic of the task paradigm. Mice were first trained to locate a baited food well in an open-field (initial learning stage). After 8 days of training, the location of the baited well was shifted to the opposite quadrant, and training proceeded for another 12 days (reversal learning stage). Mice received 10 trials each day. (B) Learning performance across days, averaged over number of mice in each group (GFP control, *n* = 8; Light-off control, *n* = 16; Light-on, *n* = 21). Error bars show SEM. GFP mice were tested in a separate cohort of mice with 5 light-on (ACh-suppressed) mice, and under-performed slightly in the initial learning stage. However their performance was similar to light-off controls in the reversal learning stage, which indicated they had successfully acquired the task. (C) Number of days taken for mice to reach and maintain an 80% success rate.

In our previous study, the sn-Plast model [20] predicted that suppressing cholinergic depression would impair reversal learning, without affecting initial place learning. To test this on a behavioural task, we implanted ChAT*ArchT mice with an optic fibre above the medial septum (S1 Fig) to target cholinergic neurons. During the task, mice received either light stimulation (light-on ACh-suppressed group, n = 21) or no light stimulation (light-off control group, n = 16). To control for the potential effects of light and heat, 8 ChAT-Cre mice were implanted and light-stimulated in the same way, but received viral injections without the optogenetic construct (GFP control group). The task was a modified dry version of the the Morris water maze task assessing spatial learning, and had two stages. At the start of each trial, two food wells were placed in the inner section of two quadrants opposite each other in a circular open-field arena; one was baited with a food reward. Mice begun each trial facing outwards, pseudo-randomly in either of the other two quadrants. In the initial learning stage, mice were trained for 8 days, with 10 trials each day, to find the baited well based on visual cues. After mice had learnt to locate the first baited well in the initial learning stage, the wells were switched to test reversal learning. In this reversal learning stage, mice had to navigate to the quadrant opposite the previously baited location for the reward, and were trained for a further 12 days (Fig 1A). Performance was measured by the percentage of correct trials per day (Fig 1B). Only mice that reached and maintained an 80% daily success rate (threshold to ascertain successful task acquisition [31, 32]) at the end of initial learning were included in the analyses. Experimental and control groups attained an 80% daily success rate within the same time frame in the initial learning stage (Fig 1C, *F*_(2,42)_ = 0.38, p = 0.69), but not in the reversal learning stage (*F*_(2,42)_ = 4.70, p = 0.014). Further analysis with Tukey’s pairwise comparison test showed that the light-on ACh-suppressed group took significantly longer to reach 80% success than the light-off (*t*_(42)_ = −2.8, *p* = 0.008, *d* = −0.92) and the GFP with light stimulation (*t*_(42)_ = −2.14, *p* = 0.04, *d* = −0.89) control groups. Notably, at the end of the reversal stage, all control mice (light-off and GFP) had attained the 80% criterion, while five light-on (ACh-suppressed) mice failed to reach this threshold, indicating much poorer reversal learning. To further quantify the behavioural effect of the cholinergic inactivation on individual mice, across successive trials and task stages, we fit a fixed-effects logistic regression to the outcome of each trial (0—no reward; 1—reward found). Regressors predicting the probability of reward discovery were experimental group type (0—light-on; 1—light-off; 2—GFP), task stage (0—initial learning; 1—reversal learning) and trial number. Interaction terms between group type and task stage, and between task stage and trial number were also included (Eq 1). The coefficients of the interaction terms for each control group by task stage were significant (light-off by stage, *z* = 4.19, *p* < 0.0001; GFP by stage, *z* = 4.14, *p* < 0.0001). This indicates that the performance difference between the control (either light-off or GFP) and light-on (ACh-suppressed) groups was greater in the reversal learning stage, compared to the between-group difference in the initial learning stage. Hence, mice receiving light-induced cholinergic inactivation learnt the location of the first baited food well as quickly as controls, but learnt the newly baited location more slowly. Taken together, these results reveal a selective impairment in reversal learning caused by the optogenetically-induced cholinergic inactivation, consistent with the predictions of our computational model [20].

### Reducing acetylcholine in the sn-Plast model qualitatively accounts for behavioural results at the group-level

**Fig 2:**
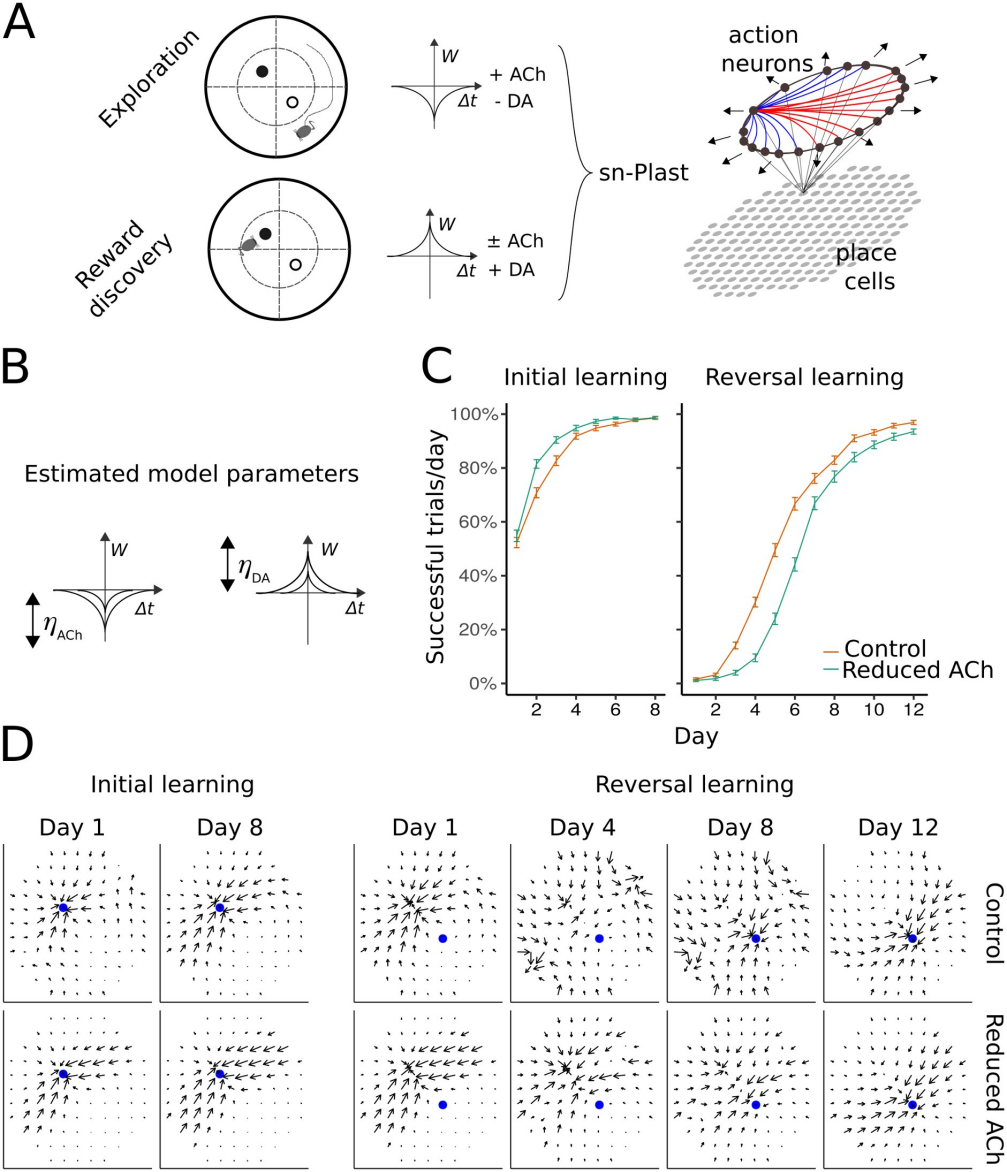
Reducing acetylcholine in the sn-Plast model qualitatively accounts for the behavioural data. (A) The sn-Plast learning rule governing synaptic weight changes in the model. STDP changes synaptic weights (*W*) as a function of the time difference between pre- and postsynaptic spikes (Δ*t*). The STDP windows are symmetric, and the sign of the weight change is determined by the neuromodulator. Acetylcholine is present during exploration, biasing STDP towards synaptic depression. When a reward is encountered, a phasic dopaminergic signal is released, which potentiates active synapses through an eligibility trace. The model consists of a one-layer network of place cells, representing the agent’s position, projecting to a ring network of recurrently connected action neurons coding for the direction taken by the agent. Connections between action neurons with similar tuning are excitatory (blue), but are inhibitory otherwise (red). The weights between place cells and action cells are modified according to the sn-Plast learning rule. (B) Learning rate parameters which control the STDP window amplitude. (C) Reducing acetylcholine in the model (*η*_*ACh*_ = 0.000345 to *η*_*ACh*_ = 0.000184, at *η*_*DA*_ = 0.00115) impairs reversal learning, reproducing learning curves similar to group performance of control and light-on mice as shown in Fig 1B. (D) Policy preference map at different stages of the task for parameters used in C. Blue filled circle indicates the location of the reward in the open maze. Vector fields (by averaging the synaptic weights from each place cell to the action neurons) represent the agent’s policy preference map across days. The effect of reducing acetylcholine in the model is evident during the early phase of reversal learning (days 4 and 8 shown); reducing acetylcholine slows unlearning of the old reward location.

To understand the synaptic mechanisms underlying the behavioural effect, we simulated the spatial learning task with our spiking neural network model endowed with sn-Plast. The sn-Plast model provides a mechanistic explanation linking the gating of plasticity by neuromodulators to changes in learning behaviour. It explicitly models how acetylcholine weakens the synapses between place and action cells that are no longer relevant to the current context, to facilitate the learning of new rewards. As in our previous computational study [20], the feedforward synaptic weights between place cells (encoding position) and action cells (encoding velocity) of the network were updated according to the sn-Plast learning rule. During exploration of the virtual environment, acetylcholine depressed active synapses. Whenever the agent located the baited food well, a phasic dopaminergic signal was delivered at the end of that trial to retroactively potentiate the synapses that participated in reward discovery, through an eligibility trace (Fig 2A). In reality, extensive training with cued rewards decreases the magnitude of the dopamine signal [33, 34]. However, for simplicity and our present purposes of testing the sn-Plast rule, we assumed that reward would consistently trigger the same amplitude of dopamine response. Future slice experiments could investigate how dopamine release with behaviourally relevant dynamics interacts with cholinergic-induced plasticity.

To simulate cholinergic neurons in mice (light-on group) being optogenetically inactivated during the task, we reduced the amount of acetylcholine, controlled by the parameter *η*_*ACh*_, released in the model. *η*_*ACh*_ scales the amplitude of the STDP window (Fig 2B), causing greater depression at higher amounts. Reducing acetylcholine in the model reproduced the selective behavioural impairment in the reversal learning stage (Fig 2C). The policy preference map (Fig 2D) shows that with less acetylcholine to depress place-action synapses that are no longer relevant, unlearning the old reward occurs more slowly.

### Heterogeneity in learning across mice

**Fig 3:**
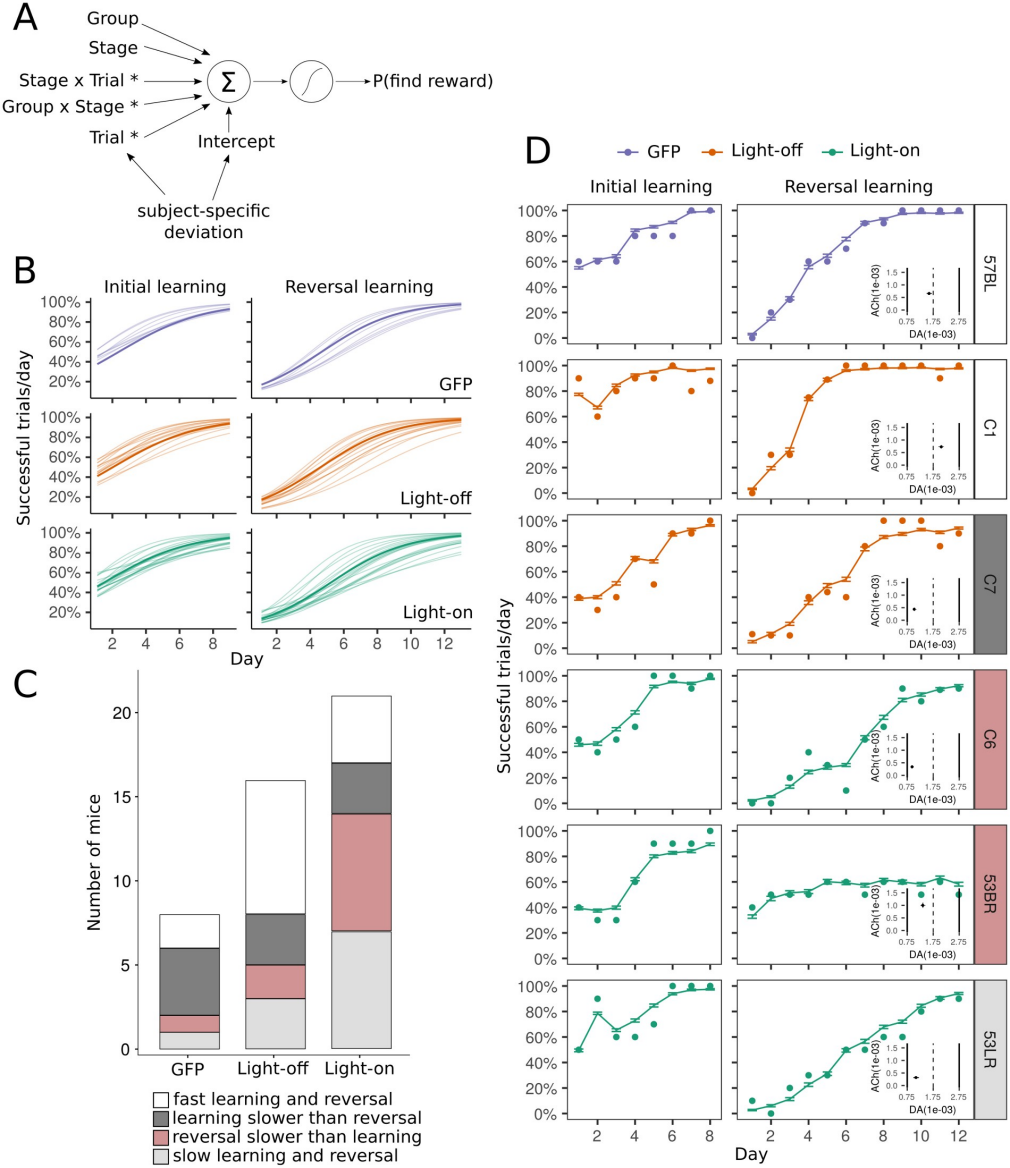
Heterogeneity in learning across mice. (A) Mixed-effects logistic regression fit to the experimental data. The five regressors used to predict the probability of a mouse locating the reward on each trial: group type (GFP, light-off or light-on), stage (initial learning or reversal learning), trial number, and interactions between the variables. Unique slopes and intercepts were estimated for all mice, which produced individual predictions shown in B. Asterisks indicate significant terms. (B) Estimated probability of individual mice locating the correct well on each day, after fitting the mixed-effects logistic regression to the data. (C) Types of learning behaviours in mice. Some mice were slower in the initial learning stage (≥ 6 days to attain 80%) than they were at reversal learning. Others were slower in the reversal learning stage (≥ 8 days to attain 80%) than they were at initial learning. There were also mice that performed consistently well (fast learning and reversal) or poorly (slow learning and reversal) across the two task stages. (D) Examples of model fits to individual mice. The sn-Plast model was fit to each mouse by comparing the RMSE of the percentage of correct trials across days between the mouse (filled circles) and the agent. This was repeated for each iteration of the model, producing 100 parameter estimates for each mouse. Simulated behavioural data across the 100 best fit estimates were then averaged to yield the performance curve (overlaid line). Error bars represent SEM. (inset) Final parameter estimate (x-coordinate, *η*_*DA*_; y-coordinate, *η*_*ACh*_). Colours of the subject labels indicate the type of learning behaviour as described in C.

To examine behavioural variability among mice, we included subject-specific intercepts and subject-specific slopes in the logistic regression fit to the experimental data (Fig 3A and S2 Fig and Eq 2). The intercept reflects the probability of success on the first trial (baseline performance), and the slope reflects the rate of learning across trials. Having subject-specific terms describes how the performance of individual mice deviates from the group-level regression line (Fig 3B). Including these terms in the logistic regression decreased the Akaike Information Criterion (fixed-effects only, 8779; with subject-specific terms, 8526) and significantly improved the fit to experimental data (*χ*^2^ = 257, *p* < 0.0001), showing that learning performance was indeed highly varied across mice. Examining the number of days taken to reach an 80% success rate in each task stage also revealed different learning patterns. While some mice performed consistently across the two task stages, others learnt the reward location in one task stage (initial or reversal learning) faster than they did in the other (Fig 3C).

We asked whether differences in neuromodulator concentrations could explain the behavioural variability in mice. *η*_*ACh*_, controlling the magnitude of cholinergic-induced depression during exploration, and *η*_*DA*_, controlling dopaminergic-induced potentiation following a reward, were the only two parameters allowed to vary in the model—all other parameters were left unchanged from previous papers [20, 35]. Although dopamine neurons were not optogenetically targeted in this experiment, *η*_*DA*_ was not constrained as we were agnostic about innate dopamine concentrations across mice, and because cholinergic activity may modulate dopamine release [36–38].

Our simulations across a wide range of parameter value combinations show that acetylcholine and dopamine affect the two task stages differently (S3 Fig). In general, overall task acquisition (initial and reversal learning) improves with more dopamine. On the other hand, reversal learning is more sensitive to acetylcholine and shows a nonlinear relationship with increasing concentration. For a given concentration of dopamine, increasing acetylcholine to a moderate level (*η*_*ACh*_/*η*_*DA*_ < 0.4) improves reversal without influencing initial learning. However, at high acetylcholine concentrations not tested in our previous work, the cumulative effect of cholinergic-induced depression over the duration of the trial impairs task acquisition, and the weights quickly saturate at their minimum limits. While acetylcholine persists throughout the trial and affects synapses at each time step, the dopaminergic signal is released only transiently after reward discovery. Hence at high acetylcholine concentrations, the dopamine signal is not enough to potentiate relevant synapses, and the agent cannot learn either reward location. These simulation results show that the time course and amounts of the two neuromodulators determine the balance between cholinergic-depression and dopaminergic-potentiation, such that acetylcholine affects learning performance in a nonlinear way. Measurements of acetylcholine *in vivo* could constrain parameter values to physiological concentrations, and establish the regime and boundary conditions in which acetylcholine operates.

To fit the model to each mouse, we performed a grid search over 51 levels of *η*_*ACh*_ × 11 levels of *η*_*DA*_, iterating the model 100 times across all parameter settings (*η*_*ACh*_, *η*_*DA*_). For each iteration, we compared learning performance between the mouse and the agent by calculating the root mean square error (RMSE) of the percentage of successful trials per day. The daily success rate, rather than the outcome of each trial, was compared because performance could fluctuate through the course of the day, over the ten trials. For each mouse, model fitting yielded a set of 100 fitted parameters and of 100 simulated behavioural data curves, which were averaged for the final parameter estimate and performance curve respectively (S4–S7 Figs).

Model parameters fit to individual mice reproduced behavioural outcomes in the experiment (S8 Fig). Comparing the number of days for agents to reach an 80% success rate (after averaging the set of 100 simulated behavioural data curves obtained for each mouse) revealed a significant learning stage-by-group interaction (*F*_(2,84)_ = 3.14, p = 0.048). Post-hoc comparisons revealed that the difference between control and light-on agents was larger during reversal learning, compared to the between-group difference during initial learning (light-off vs light-on, p = 0.037; GFP vs light-on, p = 0.05). To test for a between-group difference in performance across trials and task stages, we applied the same fixed-effects logistic regression used for the experimental data analysis (Eq 1) to each simulated behavioural dataset. In 81/100 iterations, parameters fit to light-off mice produced performance that was significantly different to that produced by parameters fit to light-on mice, and only in the reversal learning stage. This number was 71/100 comparing the GFP-control and the light-on groups. Hence, heterogeneity in neuromodulatory levels in the model can account for the diversity in learning behaviours that mice exhibit.

Contrary to our expectation, fitted acetylcholine values between control and light-on groups were not significantly different (Kruskal-Wallis test, *χ*^2^ = 1.59, p-value = 0.45). We had hypothesized that light-on mice would have lower estimated levels of *η*_*ACh*_, since cholinergic neurons were optogenetically silenced in these subjects. The absence of a detectable difference in parameters between groups could stem from either the model fitting process or having a small subject pool with high variability. However, *η*_*ACh*_ and *η*_*DA*_ could reliably be recovered from simulated data, suggesting that the lack of difference was not a problem of parameter identifiability (S9 Fig). To test how likely it was to detect between-group differences in parameter values for the subject pool size of this study, we sampled parameters from a constrained parameter space where reversal performance improves linearly with increasing acetylcholine. 16 sets of parameters were drawn for light-off mice, and 8 sets for GFP controls. 21 sets were drawn from a parameter space of reduced acetylcholine, for the light-on mice receiving cholinergic inactivation. This sampling process was repeated 1000 times (S10 Fig). In 566 of 1000 of these samples, *η*_*ACh*_ was significantly lower in the light-on group. Almost half the samples had no significant difference in *η*_*ACh*_ between groups, even after constraining the parameter space such that increasing acetylcholine enhances reversal learning. Hence fitting a larger cohort of mice might be needed to uncover meaningful differences in estimated neuromodulator values.

Certain aspects of behavioural variability, such as a marked discrepancy between initial learning and reversal learning performance, were not captured by the current model. To investigate how inconsistency across task stages influences parameter values, we fit the model to each task stage separately, to either initial learning or reversal learning data. *η*_*ACh*_ fit to the reversal stage was lower than initial learning estimates (S11 Fig) for most slow reversal learners (≥ 8 days to reach 80%), as was expected if reversal learning requires acetylcholine. Three light-on mice (“53BR”, “J2” and “R5”, S7 Fig) were the exception; they had the highest *η*_*ACh*_ estimates in the group but were the slowest at reversal learning. Despite attaining the minimum 80% criterion during initial learning, they did not show a strong preference for the old reward on the first day of reversal learning and their performance remained at chance after. In contrast, simulated agents that acquired the task would initially persist in visiting the old reward location, before unlearning it. Hence certain learning behaviours seen in mice are not yet well explained by the model. Besides between-stage variation, there were between-day fluctuations or signs of “forgetting” (“J1” and “J9”, S6 Fig; “R5”, “Q1, and “J2”, S7 Fig). Such fluctuations could reflect daily shifts between learning and unlearning, mediated by temporal changes in dopaminergic and cholinergic activity. In contrast, neuromodulator amplitudes in the current model remain constant throughout the simulated task. Finally, the model does not account for the effects of consolidation, affective factors such as motivation/impulsivity (perhaps related to velocity of the agent, S12 Fig), attention, or an initial left/right preference.

Endogenous dopamine and the type of learning behaviour also affect the comparison of estimated *η*_*ACh*_ between control and light-on groups. There was a significant effect of estimated *η*_*DA*_ (*F*_(1,41)_ = 42.56, *p* < 0.0001) on *η*_*ACh*_, suggesting an interaction between the two neuromodulators. Whether mice were slow at reversal learning also influenced *η*_*ACh*_ (*F*_(1,41)_ = 4.7, *p* = 0.036). These factors could confound between-group comparisons of parameter estimates.

It would be necessary to incorporate additional factors into the model, for *η*_*ACh*_ estimates to quantitatively reflect the effects of optogenetic silencing of cholinergic neurons. Nevertheless, the model was flexible in reproducing the between-subject variability in learning performance and the reversal learning impairment of light-on mice. Overall, our current results show a good correspondence between the sn-Plast model and experimental observations at the behavioural level.

## Discussion

Our previous experiments in mouse hippocampal slices revealed a temporally sequenced neuromodulation of STDP (sn-Plast); dopamine converted cholinergic-facilitated depression into potentiation even one-minute after the plasticity induction protocol. Based on these slice experiments alone, we had made an extrapolation from synapses to behaviour, predicting that acetylcholine-facilitated depression would aid the unlearning of old reward locations. We then simulated reward-based navigation with a computational model implementing the newly discovered sn-Plast rule and showed that acetylcholine would enhance an agent’s ability to learn a new reward [20]. The new behavioural results of this study have verified the predictions of our model. We showed here that inactivating cholinergic neurons does not affect initial learning of rewards—it impairs learning only in the second stage of the task, when the reward is shifted to a new location. The selective effect on reversal learning, rather than an overall learning deficit, suggests that acetylcholine performs a unique computational function in learning new contingencies. Specifically, acetylcholine extinguishes state-action associations that do not culminate in reward.

The sn-Plast model, conceived before the behavioural experiments, accommodated inter-individual diversity in learning behaviours and explained the performance of ACh-suppressed mice. Reducing acetylcholine in the model reproduced qualitatively the reversal learning impairment of the light-on mice at the group level. Fitting parameters to individual mice produced behavioural data that recovered the effect of silencing cholinergic neurons on reversal learning. Nonetheless, we do not suppose the model to be the only explanation for reversal learning and credit assignment, nor do we rule out other models. For example the Rescorla-Wagner model [39] also explains initial acquisition and subsequent extinction, but at a behavioural level. Our model extends observations from hippocampal slices to the open-field maze by proposing cholinergic-mediated depression as a synaptic mechanism for reversal learning. In the model, acetylcholine weakens the synapses between place and action cells that are irrelevant to the current context. This allows connections for the new reward to be strengthened by dopamine acting through an eligibility trace. Hence the sn-Plast model explicitly links the gating of neuromodulators to changes in learning behaviour, unlike a classical conditioning model. Other work modelled hippocampal memory-guided navigation [40–43], but using various versions of conventional reinforcement learning. Our results with the sn-Plast rule give new insight into hippocampus-dependent goal-directed spatial navigation.

Just two parameters—acetylcholine and dopamine—were varied for model-fitting, to avoid introducing additional assumptions beyond the scope of the slice experiments which motivated this study. Although our behavioural results showed that inactivating cholinergic neurons with optogenetics impaired reversal learning, estimated acetylcholine values between control and light-on groups did not differ significantly, possibly due to the small number of mice in this study. Hence at present the model is not able to definitively and quantitatively attribute reversal learning deficits to reduced acetylcholine in individual mice. Other factors may also be involved. Attention [30], motivation [44], innate biases and offline consolidation during sleep [45–47] all modulate learning, but are not yet controlled for in the model. Incorporating these effects into the model could more fully capture the complexity and heterogeneity of individual learning profiles in a small cohort of mice, and reflect underlying neuromodulator levels.

The dynamics and temporal profile of neuromodulatory signals present avenues for future research. We modelled neuromodulator release and activity after our slice experiment protocol, which used bath-applied acetylcholine followed by dopamine. Ambient levels of acetylcholine were simulated during agent exploration, followed by a reliable release of dopamine if the reward was found. Hence the model maintains a constant level of acetylcholine throughout the task, and reward delivery invariably produces a stable dopaminergic response. In reality, the release profiles of these neuromodulators are complex; they vary across behavioural states in the animal and play different roles. A study which implanted electrochemical biosensors for acetylcholine in mPFC and dHPC in mice recorded tonic release during maze training, and phasic release at reward delivery locations on a spatial working memory task [48]. An extension of the model could allow acetylcholine release to be modulated by familiarity with the current task demands and environment, rather than to continuously depress synapses throughout the task. The mechanism by which such a dynamic signal would coordinate learning warrants more research, as there are different forms of acetylcholine-modulated plasticity. The precise timing [49], temporal profile and concentration [14] of acetylcholine release influence the strength, duration and polarity of plasticity, through different pathways, cholinergic receptor subtypes (on pre- or post-synaptic neurons and on astrocytes), and interneuron activity [17, 50], which will have have different implications for learning. Dopaminergic activity dynamics could also be further developed in the model. It is known that the dopamine signal decreases with extensive training with cued rewards [33, 34], and is instead elicited maximally when reward is unexpected, coding for a reward prediction error [22, 23]. In previous simulations [21], we tested two feedback signals resembling the reward prediction error and compared agent performance to that under the sn-Plast learning rule. One was a dynamic reward signal which tracked reward history and was maximally activated when rewards were surprising (either encountered or omitted suddenly). The other was a negative feedback signal delivered when the expected reward was omitted, and depressed synapses retroactively through an eligibility trace. It would be possible to extend our model to study how cholinergic depression interacts with a prediction error signal, instead of the stable reward signal currently incorporated, to affect exploration and performance.

To more truly understand the orchestrated activity of acetylcholine and dopamine during behavioural learning and under the optogenetic intervention, it would be important to directly measure the two neuromodulators *in vivo*. Given that the cholinergic system modulates dopamine activity and release [36–38, 51], light-induced cholinergic inactivation could change dopamine concentrations, and consequently parameter estimates. It was difficult to make conclusions about the absolute acetylcholine values in this study, and more clarity on the operating regime of acetylcholine is needed. Our simulation results show a nonlinear relationship between acetylcholine and task performance. Increasing acetylcholine improves reversal learning, but only at low to moderate concentrations. Beyond these levels, cholinergic-depression begins to dominate synaptic changes and saturate the weights, hindering learning in both task stages. New advancements in genetically encoded fluorescent sensors for acetylcholine [52] and red-shifted sensors for dopamine [53, 54] enable simultaneous monitoring of the dynamics of the two neuromodulators during behaviour. Such technologies could provide temporally precise readouts to inform model parameters.

We believe our research contributes new understanding of the computational function of neuromodulated-plasticity [55–57] in reward learning. The work has spanned synaptic and behavioural levels, and has benefitted from the synergism between experimentation and modelling. Electrophysiological slice recordings inspired a new synaptic learning rule in a model which in turn motivated behavioural experiments. Behavioural results here have confirmed modelling predictions about the computational role of acetylcholine for new contingencies, although we cannot exclude other effects of acetylcholine. Finally, model-fitting and analyses explained individual learning behaviours, reproducing the behavioural effect of light-induced cholinergic inactivation as a result. Future work to extend the model and to monitor neuromodulator release would permit the interpretation of individual performance in terms of exact parameter estimates, completing the chain.

## Materials and methods

### Ethics statement

All animal experiments were conducted under the U.K. Animals (Scientific Procedures) Act 1986 Amendment Regulations 2012 following ethical review by the University of Cambridge Animal Welfare and Ethical Review Body (AWERB) under a Home Office project licence (PPL 7008892) and personal licences held by the authors.

### Behavioural experiments

#### Animals

Mice in the light-off and light-on groups were ChAT-Ai40D mice, the offspring of the ChAT-IRES-Cre line (Jackson Laboratories, stock #006410) crossed with the Ai40D line (Jackson Laboratories, stock #021188) bearing a Cre-dependent, enhanced GFP (eGFP)-tagged Archaerhodopsin-3 (ArchT) fusion protein. ChAT-Ai40D mice express ArchT in all cholinergic cells. For GFP-controls, ChAT-IRES-Cre mice were injected with AAV9-hsyn-GFP-WPR viral molecules. Mice were housed in polycarbonate cages of 2–10 animals and had access to food and water ad libitum, except when on food restriction during behavioural testing. Holding facilities were maintained at approximately 22 °C, 60–70% humidity, and with a 12 hour light/12 hour dark cycle.

#### Optogenetic manipulations

ArchT was excited using a yellow-green laser-light from a solid-state laser diode (561 nm; Laser 2000) that collimated into an aperture-matched fibre-optic patch cord (DoriLenses). The light output was adjusted to 26 ± 1 mW at the fibre tip. A mono fibre-optic cannula (4 mm long, 200 *μ*m diameter, 0.22 NA; Doric Lenses) was positioned above the medial septum (AP, + 1 mm; ML, 0 mm; DV, −3.55 mm) of mice (> 6 weeks old). During behavioural testing, a patch cord was used to connect the laser to the cannula via a cubic zirconia sleeve. The optic fibre positioning and expression of ArchT were confirmed using immunohistochemistry. At the end of the behavioural testing, mice were deeply anaesthetized using pentobarbital and perfused with PFA (4%). After cryopreservation in sucrose (30%), 40–60 μm slices of medial septum and hippocampus were obtained.

#### Immunohistochemistry

Sections were rinsed for 6 × 5 minutes in phosphate-buffered saline (PBS) and incubated for 1 hour in a blocking solution comprising of PBS with 0.3% (w) Triton X-100 and 5% (w) donkey serum (Abcam) containing 1% (w/v) bovine serum (Sigma). They were then incubated for 15 hours at 4 °C in blocking solution containing chicken anti-GFP (1:1000, AB13970, Abcam) and goat anti-ChAT (1:500, AB144, Milipore) antibodies. The sections were then rinsed for 6 × 5 minutes in (PBS), then incubated for 2 hours in blocking solution containing goat anti-chicken Alexa Fluor 488 (1:1000, 11039, Life technologies) and donkey anti-goat Alexa Fluor 594 (1:1000, AB150132, Abcam) at room temperature. After 6 × 5 minutes rinse, the sections were mounted in Fluoroshield with DAPI (Sigma). Fluorescence images to verify expression of the eYFP/GFP tag and to visualise ChAT labelled neurons were taken with a Leica microsystems SP8 confocal microscope using a 10× and 20× lens and acquired with Leica Microscope Imaging Software.

#### Behavioural task

The open-field maze was a green circular board of 110 cm diameter, bordered by a white 1 cm-high wall. The field was divided into quadrants which were then further divided into an outer and inner zone at 55 cm from the centre of the circular field. The testing room was lit with dimmed white light and had painted black and white visual cues around the maze. Two plastic food wells (1.5 cm high) were positioned at the centre of two opposing inner zones, but only one was baited with sweetened condensed milk food reward. Target zone designations were counterbalanced such that approximately equal proportions of each experimental group were assigned to each zone. Mice began the task facing outwards in the outer zone, either to the left or right of the baited quadrant. Each mouse received ten trials per day, for 8 and 12 consecutive days for the initial learning and reversal learning stages respectively. On the last day of the initial learning stage, the food reward was given after the mice had entered the inner section of the target quadrant as a control for mice locating reward by odour. On each day, they had five starts from the left of the target quadrant and five starts from the right in a pseudorandom order with no more than three consecutive starts from the left or right. Mice were immediately removed from the testing arena if they approached the empty well, or if they remained stationary for more than 1 minute or if they exceeded 2 minutes without solving the task. If mice reached the correct well, mice were allowed to consume the food reward and were removed from the testing arena as soon as they moved away from the food well. Between each trial, the open field was rotated 90° clockwise or anti-clockwise to ensure that intra-maze cues were not used to solve the task.

#### Behavioural analysis

To quantify the effect of optogenetic manipulation on learning performance, we fit a fixed-effects logistic regression to the outcome of each trial, *y*_*ijk*_, equal to 1 if the correct well was found and 0 otherwise. The probability of mouse *i* locating the correct well on *trial*_*j*_ (*j* = 1.1, 1.2, … for trial 1 on day 1, trial 2 on day 1) during task *stage*_*k*_ (0 for initial-learning and 1 for reversal-learning) is:

$$\begin{array}{c} \begin{array}{cc} Pr\left(y_{ijk}=1\right)=logit^{-1}( & \beta_{0}+\beta_{1}\cdot group_{i}+\beta_{2}\cdot trial_{j}+\beta_{3}\cdot stage_{k}\\ +\; & \beta_{4}\cdot trial_{j}\cdot stage_{k}+\beta_{5}\cdot group_{i}\cdot stage_{k}) \end{array} \end{array}$$ (1)

where *group*_*i*_ is the experimental group indicator, indicating whether the mouse was a GFP-control, light-off control or light-on (receiving optogenetic silencing) animal.

The parameter *β*_0_ is the overall intercept, *β*_1_ the overall effect of optogenetic light-induced silencing, *β*_2_ the change in the logit probability of finding the reward due to an additional trial, and *β*_3_ describes the overall effect of stage (switching from initial learning to reversal learning). Two-way interactions of variables *trial*_*j*_ and *group*_*i*_ with *stage*_*k*_ were included, to allow for the effects of the experimental group and trial to vary between the two stages of the experiment. The coefficient of interest was *β*_5_, associated with the *group*_*i*_ ⋅ *stage*_*k*_ interaction. If the optogenetic manipulation affected performance in the reversal stage but not in the initial learning stage, we would expect the *β*_5_ coefficient to be significant.

To describe behavioural variability among mice, we included subject-specific terms in a mixed-effects logistic regression. A unique intercept, *b*_0*i*_ was estimated for each mouse. Formally, this is a subject-specific deviation from the fixed intercept, *β*_0_. We also considered subject-specific slopes for trial, represented by *b*_4*i*_.

$$\begin{array}{c} \begin{array}{cc} Pr\left(y_{ijk}=1\right)=logit^{-1}( & \beta_{0}+\beta_{1}\cdot group_{i}+\beta_{2}\cdot trial_{j}+\beta_{3}\cdot stage_{k}\\ +\; & \beta_{4}\cdot trial_{j}\cdot stage_{k}+\beta_{5}\cdot group_{i}\cdot stage_{k}\\ +\; & b_{0i}+b_{4i}\cdot trial_{j}) \end{array} \end{array}$$ (2)

Each predictor was added sequentially and included if it was significant when the larger model (with the additional term) was compared to the smaller model using an ANOVA.

All analysis was done in R. The regressions were fit using the glm() (fixed-effects only, Eq 1) or the glmer() (mixed-effects, Eq 2) function with family = “binomial” from the lme4 package. Significance of regression coefficients were tested using the Wald test (in the summary() function). To test if experimental condition had an effect on the number of days for mice (or the simulated agent) to reach an 80% rate of success during reversal, we used the Aligned Rank Transform for nonparametric factorial ANOVAs from the ARTool package. Post-hoc pairwise comparisons were conducted using the contrasts() function from the emmeans package. Experimental data used for the analysis can be downloaded from https://github.com/gawygawy/snPlast.

### Computational modelling

#### Spiking neural network model

The navigation model is based on a one-layer network [35] and has previously been presented in [20] and [21]. All parameters were left at their original values, other than *η*_*ACh*_ and *η*_*DA*_ which were varied during model-fitting.

The place cells in the input layer code for the position of the agent in the environment. They project to the output layer of action neurons. Each one of the action neurons represents a different direction. Lateral connectivity in this layer ensures that action neurons compete with each other in a winner-take-all scheme. Their activity is then used to determine the action (i.e. direction and velocity) to take at every instant.

#### Place cells

The position of the agent at time *t* is described by the two-dimensional vector of its Cartesian coordinates, **x**(*t*). 121 place cells are aligned to the grid coordinates of a circle with radius 6.1 a.u., and the spacing between them is *σ* = 0.4. The spiking activity of place cell *i* is modelled as an inhomogeneous Poisson process, with rate $\lambda_{i}^{pc}\left(x\left(t\right)\right)$ defined as follows:

$$\begin{array}{c} \lambda_{i}^{pc}\left(x\left(t\right)\right)={\bar{\lambda }}^{pc}\exp(-\frac{||x\left(t\right)-x_{i}{\left|\right|}^{2}}{\sigma^{2}}). \end{array}$$ (3)

The firing rate $\lambda_{i}^{pc}$ is a function of the distance of the agent from the place cell centre **x**_*i*_. It is at its maximum, ${\bar{\lambda }}^{pc}=400\;\text{Hz}$, when the agent is located exactly in **x**_*i*_ and it decreases as it moves away. This mechanism simulates a place field in a 2D environment, which allows for an accurate representation of the position of the agent in the environment.

#### Action neurons

Place cells constitute the input to the network, and they all project to all action neurons with weights *w*^*feed*^. These feed-forward weights are initialized to *w*_*in*_ = 2 and bounded between *w*_*min*_ = 1 and *w*_*max*_ = 3. Action neurons are also connected with each other through synaptic weights *w*^*lat*^. The neurons are modelled using the simplified Spike Response Model [58], where the membrane potential of neuron *j* is given by:

$$\begin{array}{c} u_{j}\left(t\right)=\sum_{i}\sum_{{\bar{t}}_{i}\in F_{i}^{pc},t>{\hat{t}}_{j}}w_{ji}^{feed}\cdot \epsilon \left(t-{\bar{t}}_{i}\right)+\sum_{k,k\neq j}\sum_{{\bar{t}}_{k}\in F_{k}^{a},t>{\hat{t}}_{j}}w_{jk}^{lat}\cdot \epsilon \left(t-{\bar{t}}_{k}\right)\\ +\chi \Theta \left(t-{\hat{t}}_{j}\right)\exp(-\frac{t-{\hat{t}}_{j}}{\tau_{m}}), \end{array}$$

where *χ* = −5 mV scales the refractory period, ${\hat{t}}_{j}$ is the last postsynaptic spiking time and *ϵ* is the EPSP described by the kernel $\epsilon \left(t\right)=\frac{\epsilon_{0}}{\tau_{m}-\tau_{s}}(e^{\frac{-t}{\tau_{m}}}-e^{\frac{-t}{\tau_{s}}})\Theta \left(t\right),$ with Θ(*t*) being the Heaviside step function, *τ*_*m*_ = 20 ms, *τ*_*s*_ = 5 ms, *ϵ*_0_ = 20. $F_{i}^{pc}$ and $F_{k}^{a}$ are sets containing respectively ${\bar{t}}_{i}$ and ${\bar{t}}_{k}$, the arrival times of all spikes fired by place cell *i* and action neuron *k*. Spiking behaviour is stochastic and follows an inhomogeneous Poisson process with parameter *λ*_*j*_(*u*_*j*_(*t*)), which depends on the membrane potential at time *t*. In particular,

$$\begin{array}{c} \lambda_{j}\left(u_{j}\left(t\right)\right)=\lambda_{0}\exp(\frac{u_{j}\left(t\right)-\theta }{\Delta u}), \end{array}$$ (4)

where *λ*_0_ = 60 Hz is the maximum firing rate, Δ*u* = 2mV regulates randomness of the spiking behaviour and *θ* = 16 mV is a constant parameter.

Action neurons represent different directions in the Cartesian plane. Specifically, each action neuron *j* represents direction **a**_*j*_, where **a**_*j*_ = *a*_0_(sin(*θ*_*j*_), cos(*θ*_*j*_)), with $\theta_{j}=\frac{2j\pi }{N}$, *N* = 40 and *a*_0_ = 0.08. The lateral connectivity between action neuron *k* and action neuron *j* is defined as follows

$$\begin{array}{c} w_{jk}^{lat}=\frac{w_{-}}{N}+w_{+}\frac{f(j,k)}{N}, \end{array}$$ (5)

where *w*_−_ = −300, *w*_+_ = 100 and *f* is a lateral connectivity function, which is symmetric, positive and increases monotonically with the similarity of the actions. In particular, *f*(*j*, *k*) = (1 − *δ*_*jk*_)*e*^*ψ*cos(*θ*_*j*_ − *θ*_*k*_)^, with *ψ* = 20. Neurons therefore excite each other when they have a similar tuning, and depress otherwise. This ensures that only a few similarly tuned action neurons are active at any given time, making the trajectory of the agent smooth and consistent.

#### Action selection

The action selection process determines the decision to take, based on the firing rates of the action neurons. The activity of action neuron *j* is approximated by filtering spike train *Y*_*j*_ with kernel *γ*:

$$\begin{array}{c} \rho_{j}\left(t\right)=\left(Y_{j}\circ \gamma \right)\left(t\right), \end{array}$$ (6)

where $Y_{j}=\sum_{{\bar{t}}_{j}\in F_{j}^{a}}\delta \left(t-{\bar{t}}_{j}\right)$ and $\gamma \left(t\right)=\frac{e^{\frac{-t}{\tau_{\gamma }}}-e^{\frac{-t}{\nu_{\gamma }}}}{\tau_{\gamma }-\nu_{\gamma }}\Theta \left(t\right),$ with *τ*_*γ*_ = 50 ms and *ν*_*γ*_ = 20 ms. Actions are taken continuously, at every timestep *t*. The action selection process thus determines **a**(*t*), the action to take at time *t*.

If each action neuron *j* represents direction **a**_*j*_ and has an estimated firing rate *ρ*_*j*_(*t*), then the action **a**(*t*) is the average of all the directions encoded, weighted by their respective firing rates

$$\begin{array}{c} a\left(t\right)=\frac{1}{N}\sum_{j}\rho_{j}\left(t\right)a_{j}, \end{array}$$ (7)

where *N* = 40 is the total number of action neurons. This decision making mechanism allows the agent to move in any direction, making the action space effectively continuous.

#### Navigation

Once action **a**(*t*) has been determined, the update for the position of the agent is

$$\begin{array}{c} \Delta x\left(t\right)=\{\begin{array}{cc} a(t), & \text{if}\;\text{x}\text{(t+1)}\;\text{within}\;\text{the}\;\text{boundaries}.\\ a\left(t\right)-2\left(a\left(t\right)\cdot \frac{x(t)}{\left||x(t)|\right|}\right)\frac{x(t)}{\left||x(t)|\right|} & \;\text{otherwise}. \end{array} \end{array}$$

The agent therefore normally moves with instantaneous velocity **a**(*t*). If the agent encounters the boundary of the arena, its direction vector is reflected in the opposite direction. To avoid large boundary effects, the feed-forward weights between place cells on the boundaries and action neurons that code for a direction **a**_*j*_ outside of the arena are set to zero.

The agent is free to explore the environment for a maximum duration of *T*_*max*_ = 15 s. If it finds the reward at a time *t*_*rew*_ < *T*_*max*_, the trial is terminated earlier, precisely at time *t* = *T*_*rew*_ + 300 ms. The extra time mimics consummatory behavior, navigation is thus paused during this interval (i.e. place cells activity is set to zero). If the agent encounters the wrong well, the trial is terminated immediately. The effect of the inter-trial interval is modelled by resetting all activity in the action and place cells, but not in the weights.

#### Simulation of the open-field spatial learning task

The model was run for 8 × 10 = 80 trials to simulate training for ten trials/day over 8 days of initial place learning, and for 12 × 10 = 120 trials to simulate ten trials/day over 12 days of reversal learning. The two well locations were simulated as two circles placed opposite to each other in the inner quadrants of the circular field centered at *c*_1_ = (−0.43, 0.43) and *c*_2_ = (0.43, −0.43) with radius *r*_1_ = 0.3. For the first 80 trials, *c*_1_ was the location of the baited well, and in the next 120 trials, the baited well was at *c*_2_. The agent began each trial from the outer quadrants of the field, to the left (-1.6, -1.2) or right (1.6, 1.2) of the baited quadrants in a random order.

#### Sequentially neuromodulated plasticity (sn-Plast)

The synaptic weights between place cells and action neurons play a fundamental role in defining a policy for the agent. Plasticity is essential for the agent to learn to navigate the open field and is implemented in a way that follows the experimental results presented in Brzosko et al. 2015 and 2017 [16, 20]. The synaptic changes combine the modified STDP rule and an eligibility trace that allows for delayed updates. The total weight update is

$$\begin{array}{c} \Delta w_{ji}\left(t\right)=\eta A((\sum_{{\bar{t}}_{i}\in F_{i}^{pc}}\sum_{{\bar{t}}_{j}\in F_{j}^{a}}W({\bar{t}}_{j}-{\bar{t}}_{i}))\circ \psi )\left(t\right), \end{array}$$ (8)

where *η* is the learning rate, *A* emulates the effect of the different neuromodulators, *W* is the STDP window and *ψ* is the eligibility trace. $F_{i}^{pc}$ and $F_{j}^{a}$ are sets containing respectively ${\bar{t}}_{i}$ and ${\bar{t}}_{j}$, the arrival times of all spikes fired by place cell *i* and action neuron *j*.

The basic STDP window is

$$\begin{array}{c} W\left(x\right)=e^{-\frac{\left|x\right|}{\tau }}, \end{array}$$ (9)

with *τ* = 10 ms. This function is always symmetric and positive, but the sign of the final weight change is determined by the neuromodulators at the synapse:

$$\begin{array}{cc} A= & \left\{ \begin{array}{cc} -1 & \;\text{-DA,}\;\text{+ACh}\\ 0 & \;\text{-DA,}\;\text{-ACh}\\ 1 & \;\text{+DA},\pm \text{ACh}. \end{array}\right. \end{array}$$ (10)

Dopamine is assumed to be released simultaneously in all synapses whenever a reward is delivered. All weight changes are gated by neuromodulation (*A* = 0 when all neuromodulators are absent). The learning rate *η* also depends on neuromodulators:

$$\begin{array}{cc} \eta = & \left\{ \begin{array}{cc} \eta_{\text{ACh}} & \;\text{-DA,}\;\text{+ACh}\;\\ 0 & \;\text{-DA,}\;\text{-ACh}\;\\ \eta_{\text{DA}} & \;\text{+DA},\pm \text{ACh}. \end{array}\right. \end{array}$$ (11)

The weight change due to STDP is convoluted with an eligibility trace *ψ*, modelled as an exponential decay $\psi \left(t\right)=e^{-\alpha \frac{t}{\tau_{e}}}\Theta \left(t\right)$, with *τ*_*e*_ = 2 s and

$$\begin{array}{cc} \alpha = & \left\{ \begin{array}{cc} 1 & \;\text{+DA}\\ 0 & \;\text{-DA}. \end{array}\right. \end{array}$$ (12)

The eligibility trace keeps track of the active synapses and allows for a delayed update of the synaptic strength. Variable *α* in the exponent acts as a flag and ensures that the eligibility trace is active with dopamine only (*α* = 1).

#### Grid search

We varied *η*_*DA*_ from 7.5 × 10^−4^ to 2.75 × 10^−3^ in steps of 2 × 10^−4^. At every level of *η*_*DA*_, *η*_*ACh*_ was varied such that the ratio of *η*_*ACh*_:*η*_*DA*_ increased from 0 to 1, in steps of 0.02. This produced 561 combinations of the 2 parameters. We ran 100 iterations at each parameter setting.

#### Model fitting and parameter estimation

The fit of the model for a particular combination of parameter values at each iteration, $\theta_{n}=\left(\eta_{n}^{ACh},\eta_{n}^{DA}\right)$, was quantified using the RMSE, comparing the percentage of successful trials per day. The best fit parameters were averaged across the 100 iterations to yield estimates of *η*_*ACh*_ and *η*_*DA*_ for each mouse.

## Supporting information

S1 Fig Immunostaining of light-activated archaerhodopsin (ArchT) in a coronal slice of the medial septum. (A) Selective expression of ArchT-eGFP in cholinergic neurons in ChAT-Ai40D (choline acetyltransferase-Cre transgenic line) mice. DAPI (blue), ChAT (red) and eGFP-(green)-positive immunostaining. Scale bar: 40μm. (B) Histological reconstructions of the location of the implanted optic fibers.
(TIF)

S2 Fig Unique intercepts and slopes estimated for each mouse by fitting a logistic regression to the behavioural data. A mixed effects logistic regression (Fig 3A; Eq 2) was used to predict the probability of a mouse locating the reward on each trial. For each mouse, a unique intercept (baseline performance on day 1) and slope (overall rate of learning across trials) were estimated. Shown here are the subject-specific deviations from the group-level intercepts and slopes.
(TIF)

S3 Fig Effect of acetylcholine and dopamine levels on learning behaviour in the model. (A) Heat map showing the number of days to reach an 80% success rate during the initial learning and reversal learning stages, for different combinations of acetylcholine (shown as a ratio of *η*_*ACh*_/*η*_*DA*_ at each level of *η*_*DA*_) and dopamine values. Darker shades indicate poorer performance. Note how for *η*_*ACh*_/*η*_*DA*_ < 0.4, increasing *η*_*ACh*_ quickens reversal learning, with little effect on initial learning. (B) Predicted probability of the agent locating the correct well during initial learning and reversal learning, at different levels of acetylcholine. At low levels of acetylcholine, the lack of cholinergic-facilitated depression causes the agent to persist in a previously learnt path and slows reversal learning. On the other hand, very strong cholinergic depression relative to dopaminergic potentiation hinders the acquisition of the task as relevant synapses are only weakly potentiated, and the agent learns poorly.
(TIF)

S4 Fig Estimated *η*_*ACh*_ and *η*_*DA*_ in mice from model-fitting. Parameter estimates (bootstrapped mean and confidence intervals) of mouse-specific acetylcholine and dopamine levels for the three groups, overlaid on the heatmap of simulated performance as shown in S3 Fig.
(TIF)

S5 Fig Model fits to individual mice in the control GFP group. Model fits to individual mice in the GFP group. Each panel displays data from a single mouse. Panels are ordered according to the number of days taken to reach 80% performance during reversal, from the fastest (top left) to slowest (bottom right) performers. Points in each panel are the percentage of correct trials across days (8 days of initial learning followed by 12 of reversal learning). Overlaid is the model fit (line)—performance of the agent (averaging over 100 fits for each mouse). Error bars represent SEM. (inset) Parameter estimate (x-coordinate, *η*_*DA*_; y-coordinate, *η*_*ACh*_) when the model was fit either to initial learning (grey shaded area) or to reversal learning data. Lines connecting the estimates show how the values of neuromodulators change across the two task stages.
(TIFF)

S6 Fig Model fits to individual mice in the control light-off group. Model fits to individual mice in the light-off group. Each panel displays data from a single mouse. Panels are ordered according to the number of days taken to reach 80% performance during reversal, from the fastest (top left) to slowest (bottom right) performers. Points in each panel are the percentage of correct trials across days (8 days of initial learning followed by 12 of reversal learning). Overlaid is the model fit (line)—performance of the agent (averaging over 100 fits for each mouse). Error bars represent SEM. (inset) Parameter estimate (x-coordinate, *η*_*DA*_; y-coordinate, *η*_*ACh*_) when the model was fit either to initial learning (grey shaded area) or to reversal learning data. Lines connecting the estimates show how the values of neuromodulators change across the two stages of the task. Note how mouse “J9” did not show a strong preference for the old reward location on the first day of reversal, and was slow in reversal learning, but had high estimated *η*_*ACh*_.
(TIFF)

S7 Fig Model fits to mice receiving optogenetic inactivation of cholinergic neurons (light-on, ACh-suppressed). Model fits to individual mice in the light-on group. Each panel displays data from a single mouse. Panels are ordered according to the number of days taken to reach 80% performance during reversal, from the fastest (top left) to slowest (bottom right) performers. Points in each panel are the percentage of correct trials across days (8 days of initial learning followed by 12 of reversal learning). Overlaid is the model fit (line)—performance of the agent (averaging over 100 fits for each mouse). Error bars represent SEM. (inset) Parameter estimate (x-coordinate, *η*_*DA*_; y-coordinate, *η*_*ACh*_) when the model was fit either to initial learning (grey shaded area) or to reversal learning data. Lines connecting the estimates show how the values of neuromodulators change across the two stages of the task. Subjects “J2”, “53BR”, and “R5” did not show a strong preference for the old reward location on the first day of reversal, and were unable to learn the second reward location. Estimated *η*_*ACh*_ in these subjects was high despite the poor reversal learning performance.
(TIFF)

S8 Fig Behaviour reproduced from parameters fitted to individual mice. The model was fit to individual mice by selecting the set of parameters with the lowest RMSE for each iteration of model simulation. Parameters and agent behaviour (percentage of successful trials per day) were averaged across 100 iterations to yield final estimates for each mouse. This process of model-fitting reproduced the two behavioural measures in the experiment. (A) Successful trials across days averaged over number of fitted subjects in each group. As described in the main text, applying the logistic regression from the experimental data analysis revealed a selective effect of group-type only in the reversal learning stage (GFP vs light-on, 71 out of 100 model iterations; light-off vs light-on, 81 out of 100 model iterations). (B) Comparison of the number of days to attain and maintain an 80% success rate. The difference between control and light-on groups was larger in the reversal stage compared to the between-group differences in the initial learning stage.
(TIF)

S9 Fig Parameter recovery. To establish parameter identifiability, we fit the model to 200 agents simulated from randomly-drawn parameter sets in the grid search. The estimated parameters are plotted against the values of the true parameters. Dotted line is the line of unity.
(TIF)

S10 Fig Testing group differences in acetylcholine values in simulated draws. (A) Sets of parameters for the number of mice in the control groups (GFP, 8; light-off, 16) were drawn from the parameter space bordered in the solid black outline. 21 sets for light-on mice were drawn from an area (dashed outline) with reduced acetylcholine. (B) Group differences in parameter values were tested using the Kruskal-wallis test. Shown here are the results for 10 samples.
(TIF)

S11 Fig Parameter estimates across initial and reversal learning. Here the model was fitted separately to data in each task stage, to see how well acetylcholine and dopamine values correlate across initial (points in grey area) and reversal learning. For most slow reversers (bottom panels), there appears to be a reduction in acetylcholine across initial and reversal learning. However, three light-on mice which did not show a strong preference for the old reward location on the first day of the reversal had high estimated *η*_*ACh*_. These trends are also shown matched to individual mice in the inset panels of S5–S7 Figs.
(TIF)

S12 Fig Effect of agent speed on performance. The effect of increasing agent speed on initial learning and reversal learning, at different acetylcholine levels, when *η*_*DA*_ = 0.00135.
(TIF)
