# Supplementary figures and images for: The functional role of sequentially neuromodulated synaptic plasticity in behavioural learning
Source: PLoS Comput Biol. 2021 Jun 10;17(6):e1009017. doi: 10.1371/journal.pcbi.1009017 (PMC8192019; doi:10.1371/journal.pcbi.1009017)

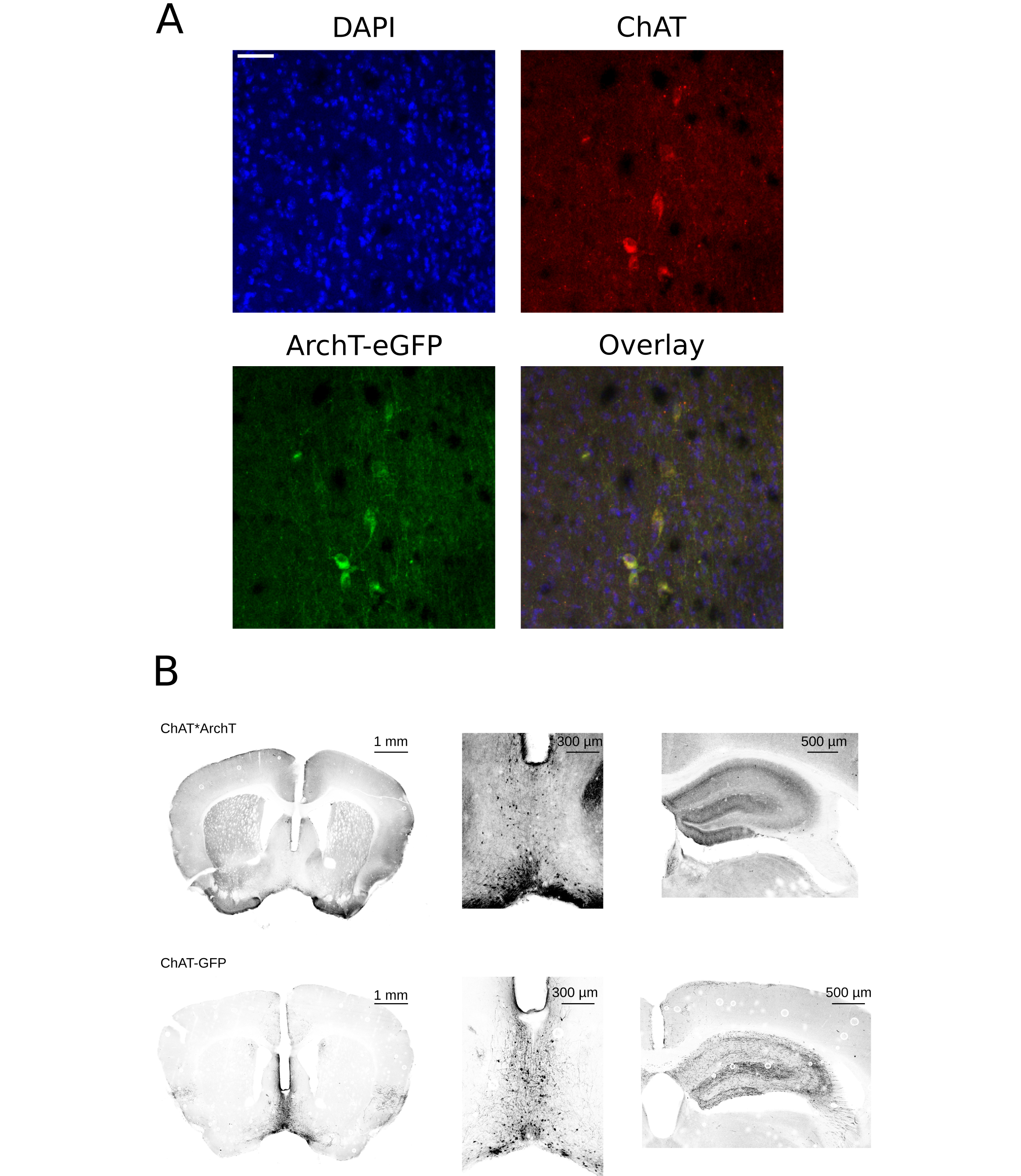

Supplement: S1 Fig — (A) Selective expression of ArchT-eGFP in cholinergic neurons in ChAT-Ai40D (choline acetyltransferase-Cre transgenic line) mice. DAPI (blue), ChAT (red) and eGFP-(green)-positive immunostaining. Scale bar: 40μm. (B) Histological reconstructions of the location of the implanted optic fibers. (TIF) [file pcbi.1009017.s001.tif]

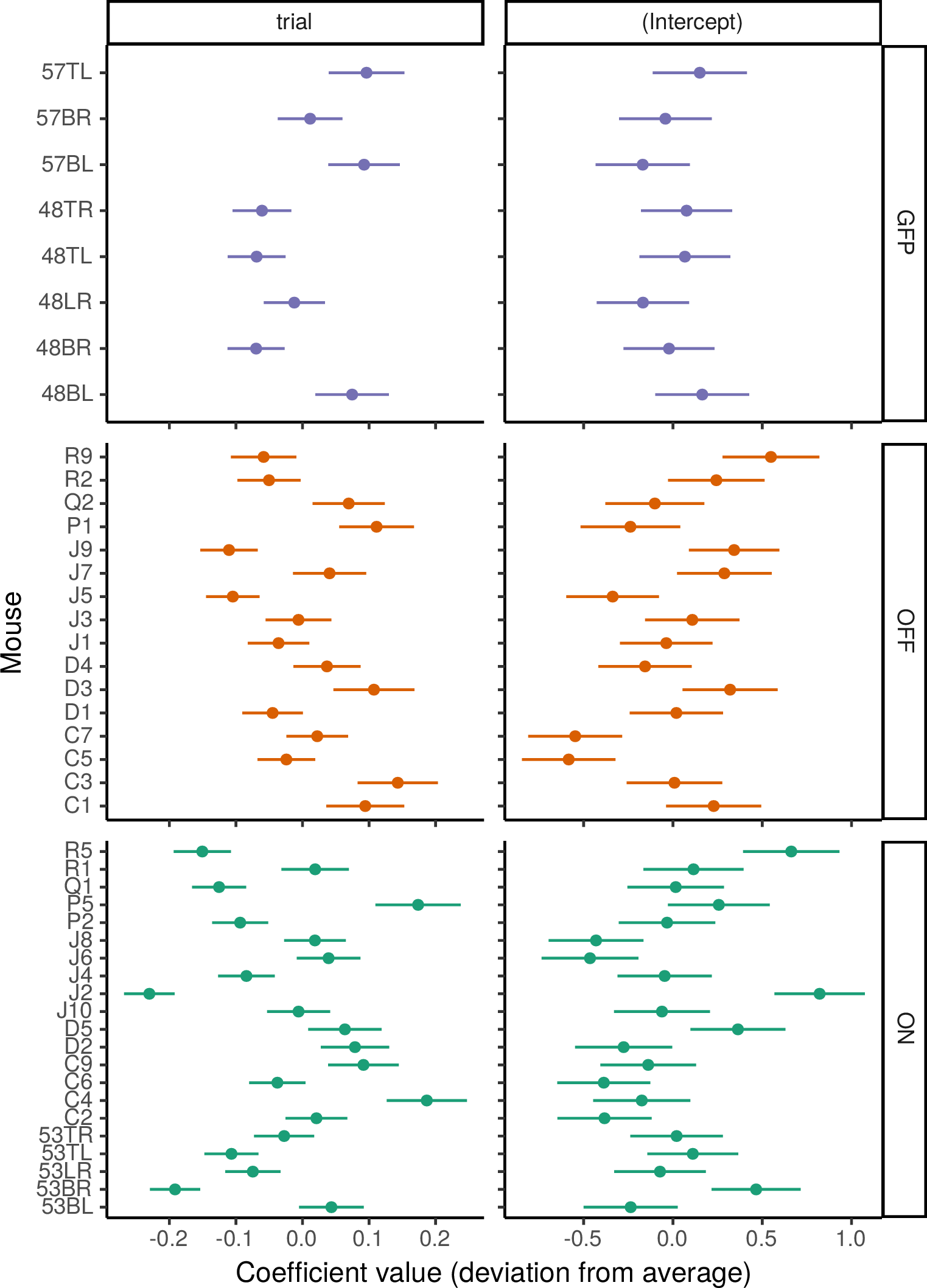

Supplement: S2 Fig — A mixed effects logistic regression (Fig 3A; Eq 2) was used to predict the probability of a mouse locating the reward on each trial. For each mouse, a unique intercept (baseline performance on day 1) and slope (overall rate of learning across trials) were estimated. Shown here are the subject-specific deviations from the group-level intercepts and slopes. (TIF) [file pcbi.1009017.s002.tif]

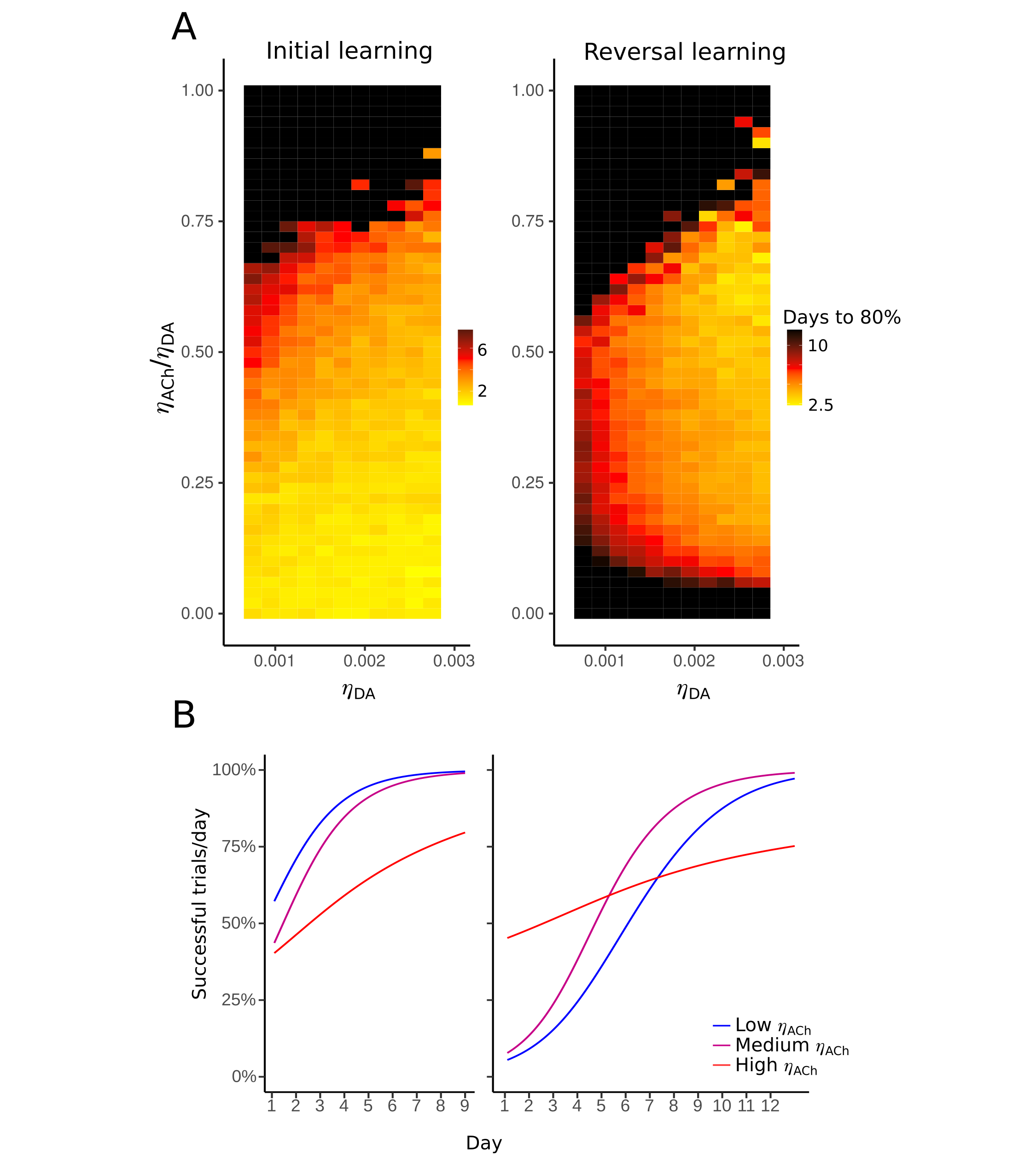

Supplement: S3 Fig — (A) Heat map showing the number of days to reach an 80% success rate during the initial learning and reversal learning stages, for different combinations of acetylcholine (shown as a ratio of ηACh/ηDA at each level of ηDA) and dopamine values. Darker shades indicate poorer performance. Note how for ηACh/ηDA < 0.4, increasing ηACh quickens reversal learning, with little effect on initial learning. (B) Predicted probability of the agent locating the correct well during initial learning and reversal learning, at different levels of acetylcholine. At low levels of acetylcholine, the lack of cholinergic-facilitated depression causes the agent to persist in a previously learnt path and slows reversal learning. On the other hand, very strong cholinergic depression relative to dopaminergic potentiation hinders the acquisition of the task as relevant synapses are only weakly potentiated, and the agent learns poorly. (TIF) [file pcbi.1009017.s003.tif]

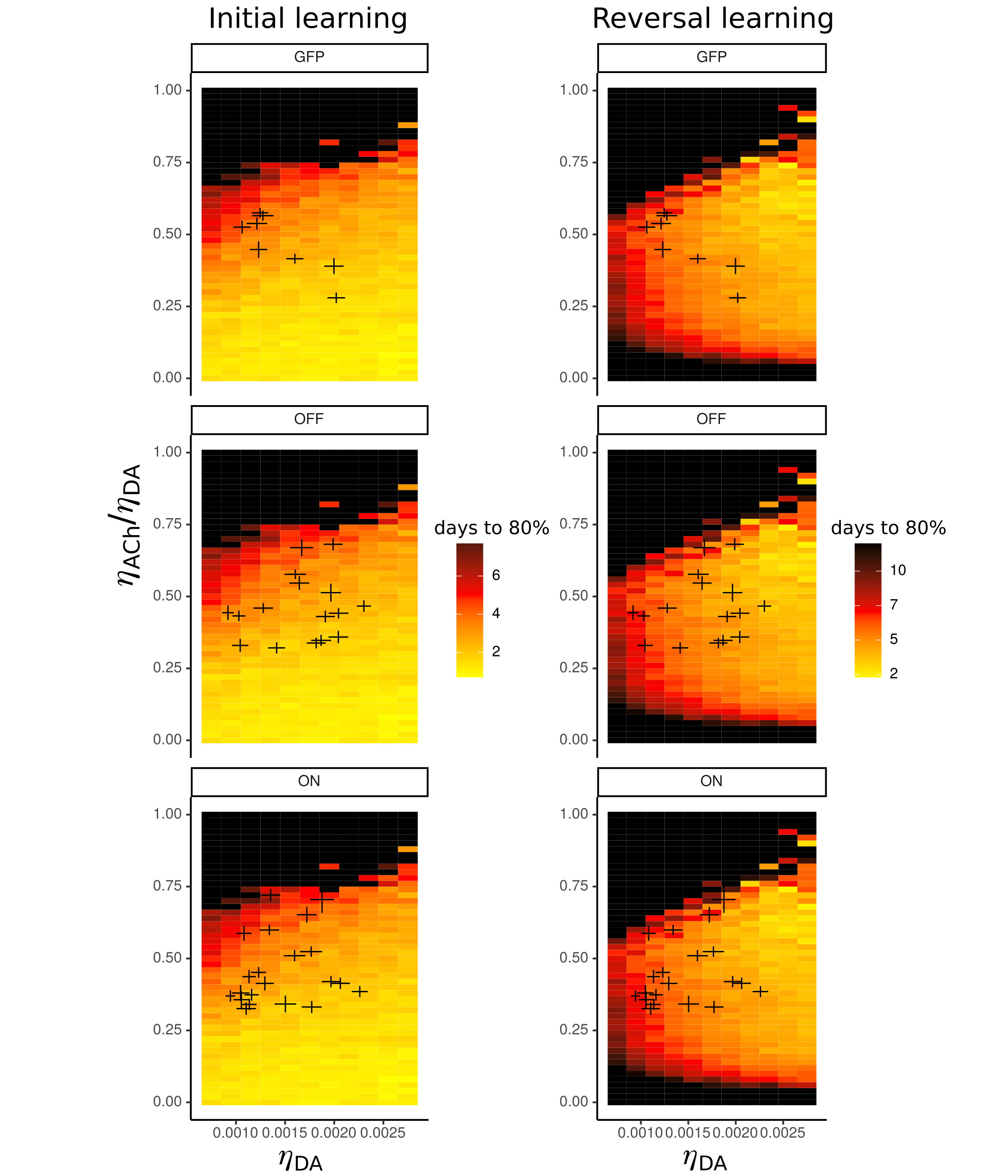

Supplement: S4 Fig — Parameter estimates (bootstrapped mean and confidence intervals) of mouse-specific acetylcholine and dopamine levels for the three groups, overlaid on the heatmap of simulated performance as shown in S3 Fig. (TIF) [file pcbi.1009017.s004.tif]

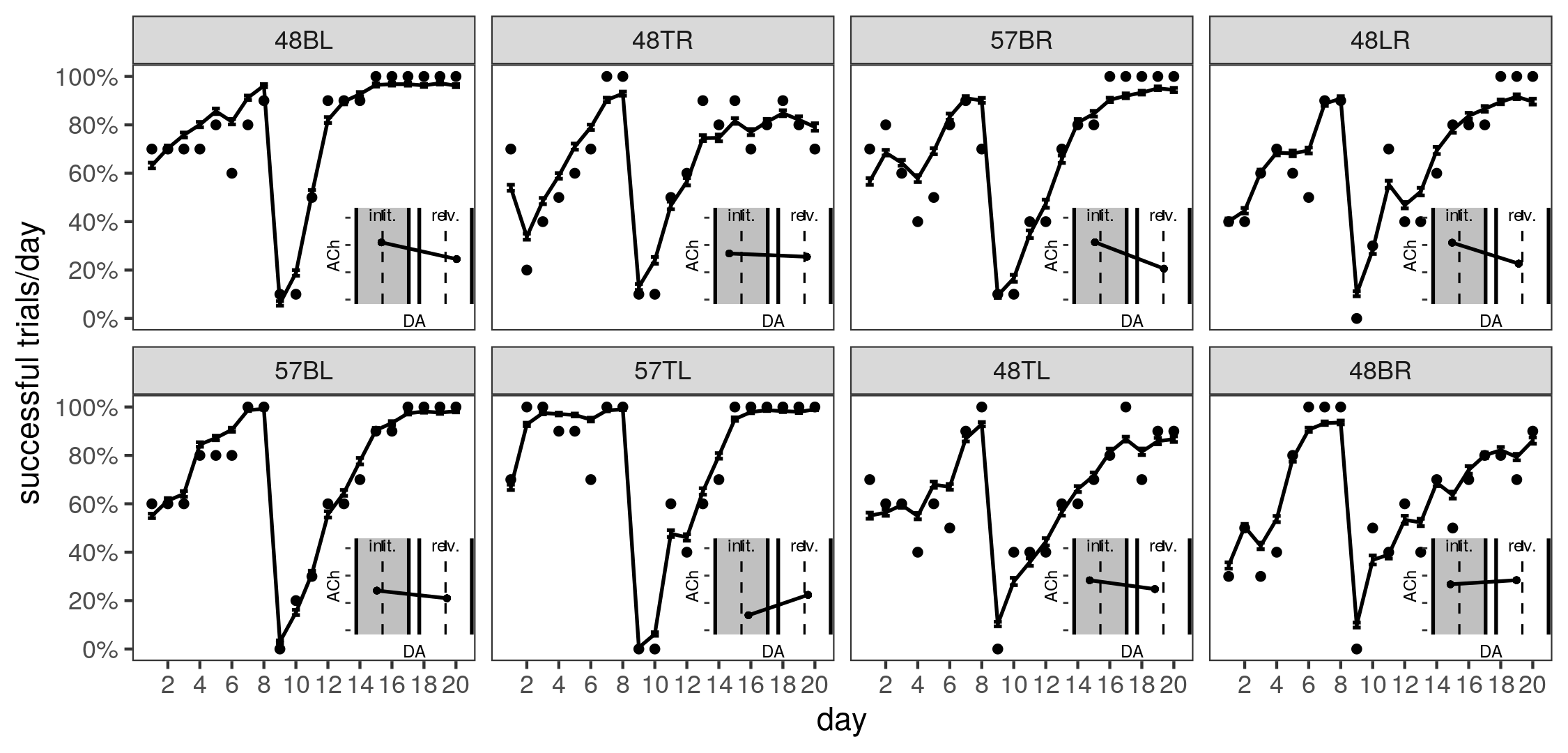

Supplement: S5 Fig — Model fits to individual mice in the GFP group. Each panel displays data from a single mouse. Panels are ordered according to the number of days taken to reach 80% performance during reversal, from the fastest (top left) to slowest (bottom right) performers. Points in each panel are the percentage of correct trials across days (8 days of initial learning followed by 12 of reversal learning). Overlaid is the model fit (line)—performance of the agent (averaging over 100 fits for each mouse). Error bars represent SEM. (inset) Parameter estimate (x-coordinate, ηDA; y-coordinate, ηACh) when the model was fit either to initial learning (grey shaded area) or to reversal learning data. Lines connecting the estimates show how the values of neuromodulators change across the two task stages. (TIFF) [file pcbi.1009017.s005.tiff]

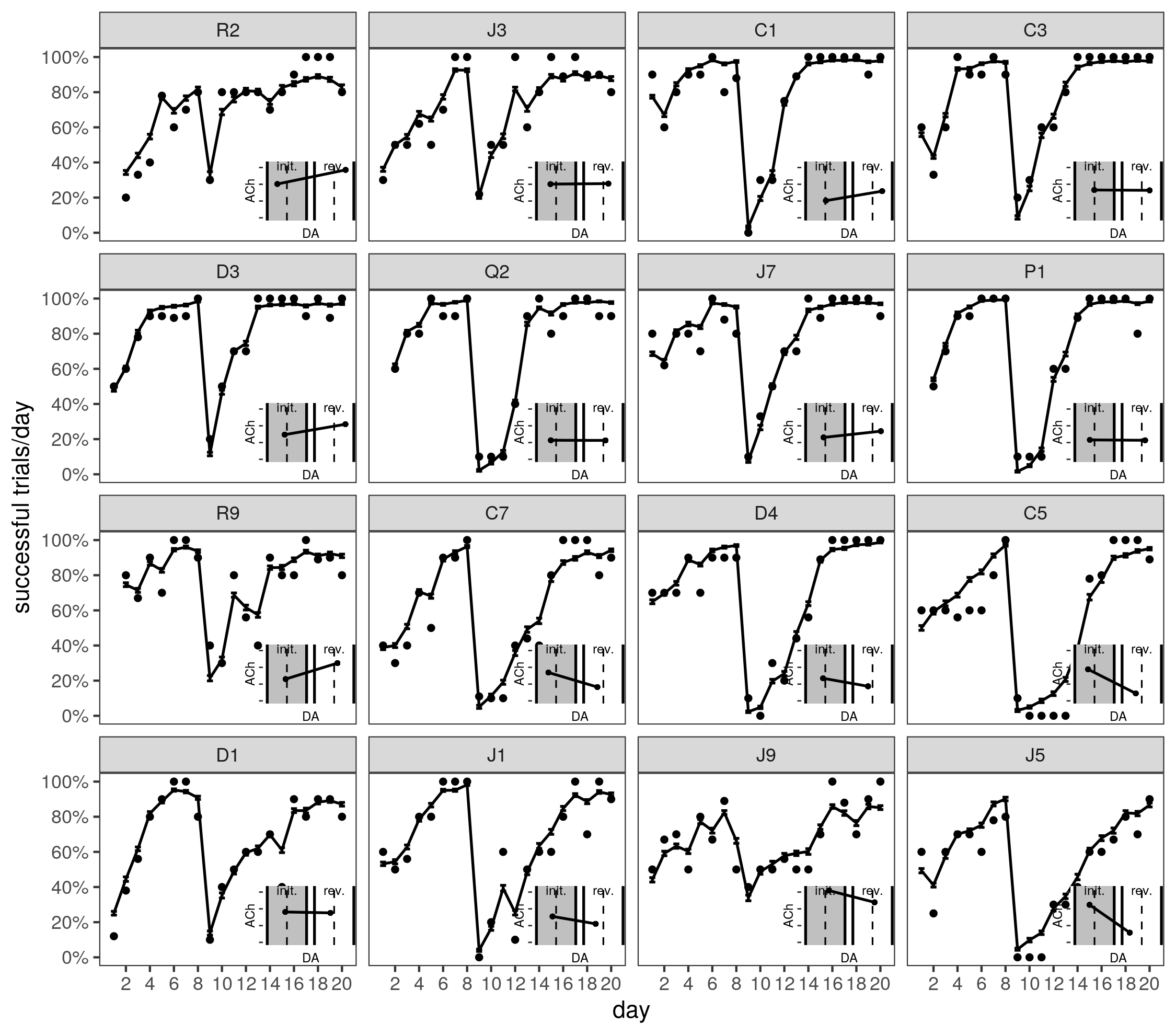

Supplement: S6 Fig — Model fits to individual mice in the light-off group. Each panel displays data from a single mouse. Panels are ordered according to the number of days taken to reach 80% performance during reversal, from the fastest (top left) to slowest (bottom right) performers. Points in each panel are the percentage of correct trials across days (8 days of initial learning followed by 12 of reversal learning). Overlaid is the model fit (line)—performance of the agent (averaging over 100 fits for each mouse). Error bars represent SEM. (inset) Parameter estimate (x-coordinate, ηDA; y-coordinate, ηACh) when the model was fit either to initial learning (grey shaded area) or to reversal learning data. Lines connecting the estimates show how the values of neuromodulators change across the two stages of the task. Note how mouse “J9” did not show a strong preference for the old reward location on the first day of reversal, and was slow in reversal learning, but had high estimated ηACh. (TIFF) [file pcbi.1009017.s006.tiff]

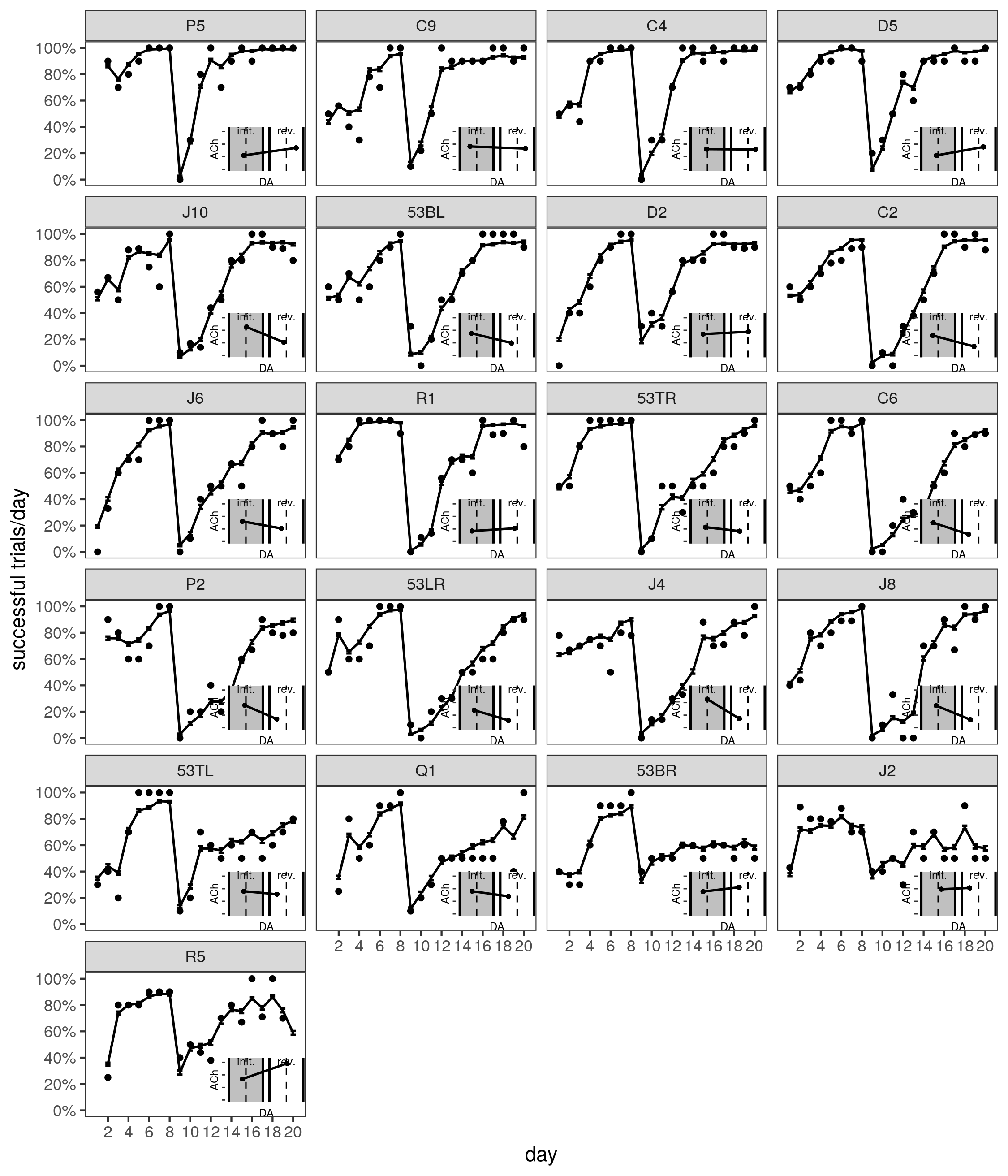

Supplement: S7 Fig — Model fits to individual mice in the light-on group. Each panel displays data from a single mouse. Panels are ordered according to the number of days taken to reach 80% performance during reversal, from the fastest (top left) to slowest (bottom right) performers. Points in each panel are the percentage of correct trials across days (8 days of initial learning followed by 12 of reversal learning). Overlaid is the model fit (line)—performance of the agent (averaging over 100 fits for each mouse). Error bars represent SEM. (inset) Parameter estimate (x-coordinate, ηDA; y-coordinate, ηACh) when the model was fit either to initial learning (grey shaded area) or to reversal learning data. Lines connecting the estimates show how the values of neuromodulators change across the two stages of the task. Subjects “J2”, “53BR”, and “R5” did not show a strong preference for the old reward location on the first day of reversal, and were unable to learn the second reward location. Estimated ηACh in these subjects was high despite the poor reversal learning performance. (TIFF) [file pcbi.1009017.s007.tiff]

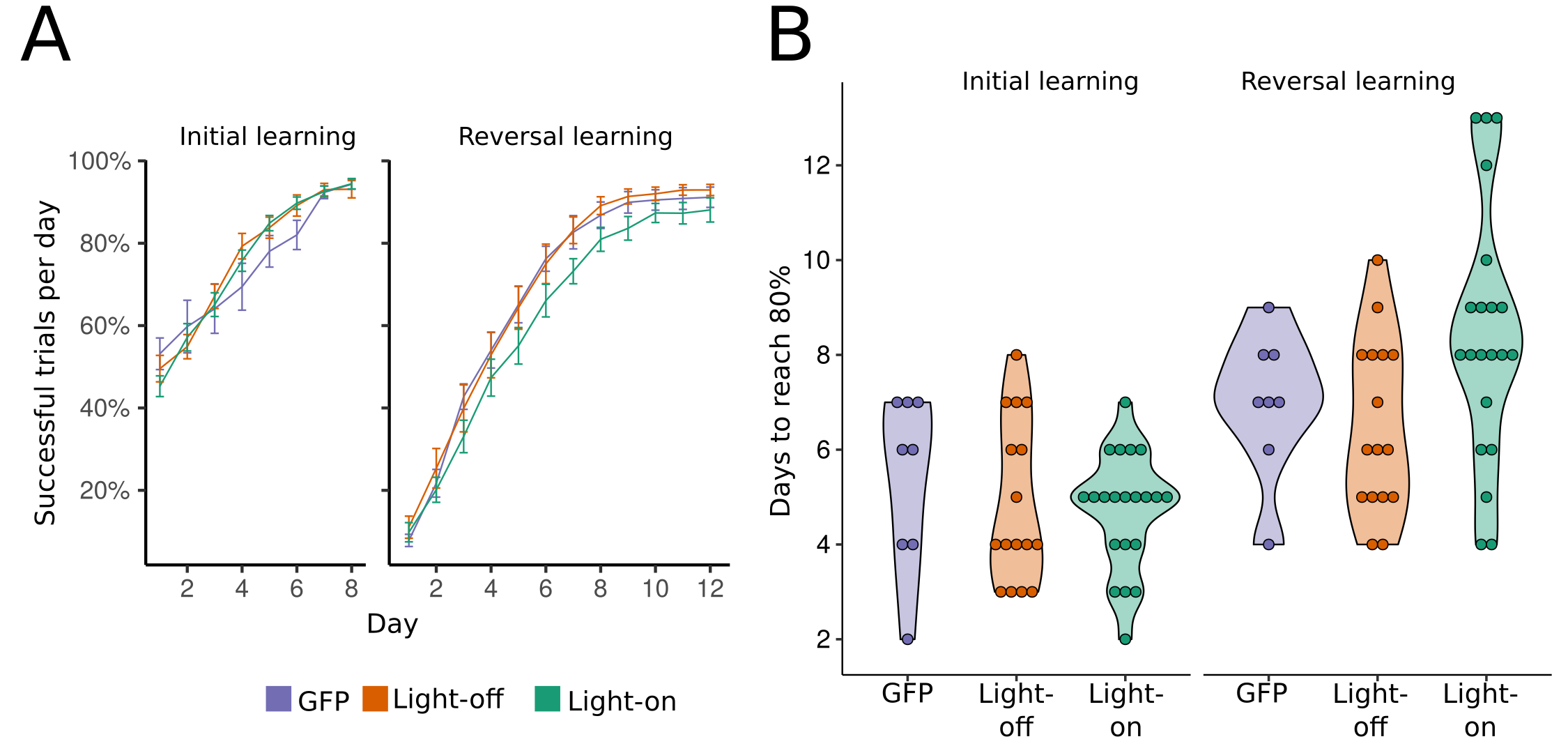

Supplement: S8 Fig — The model was fit to individual mice by selecting the set of parameters with the lowest RMSE for each iteration of model simulation. Parameters and agent behaviour (percentage of successful trials per day) were averaged across 100 iterations to yield final estimates for each mouse. This process of model-fitting reproduced the two behavioural measures in the experiment. (A) Successful trials across days averaged over number of fitted subjects in each group. As described in the main text, applying the logistic regression from the experimental data analysis revealed a selective effect of group-type only in the reversal learning stage (GFP vs light-on, 71 out of 100 model iterations; light-off vs light-on, 81 out of 100 model iterations). (B) Comparison of the number of days to attain and maintain an 80% success rate. The difference between control and light-on groups was larger in the reversal stage compared to the between-group differences in the initial learning stage. (TIF) [file pcbi.1009017.s008.tif]

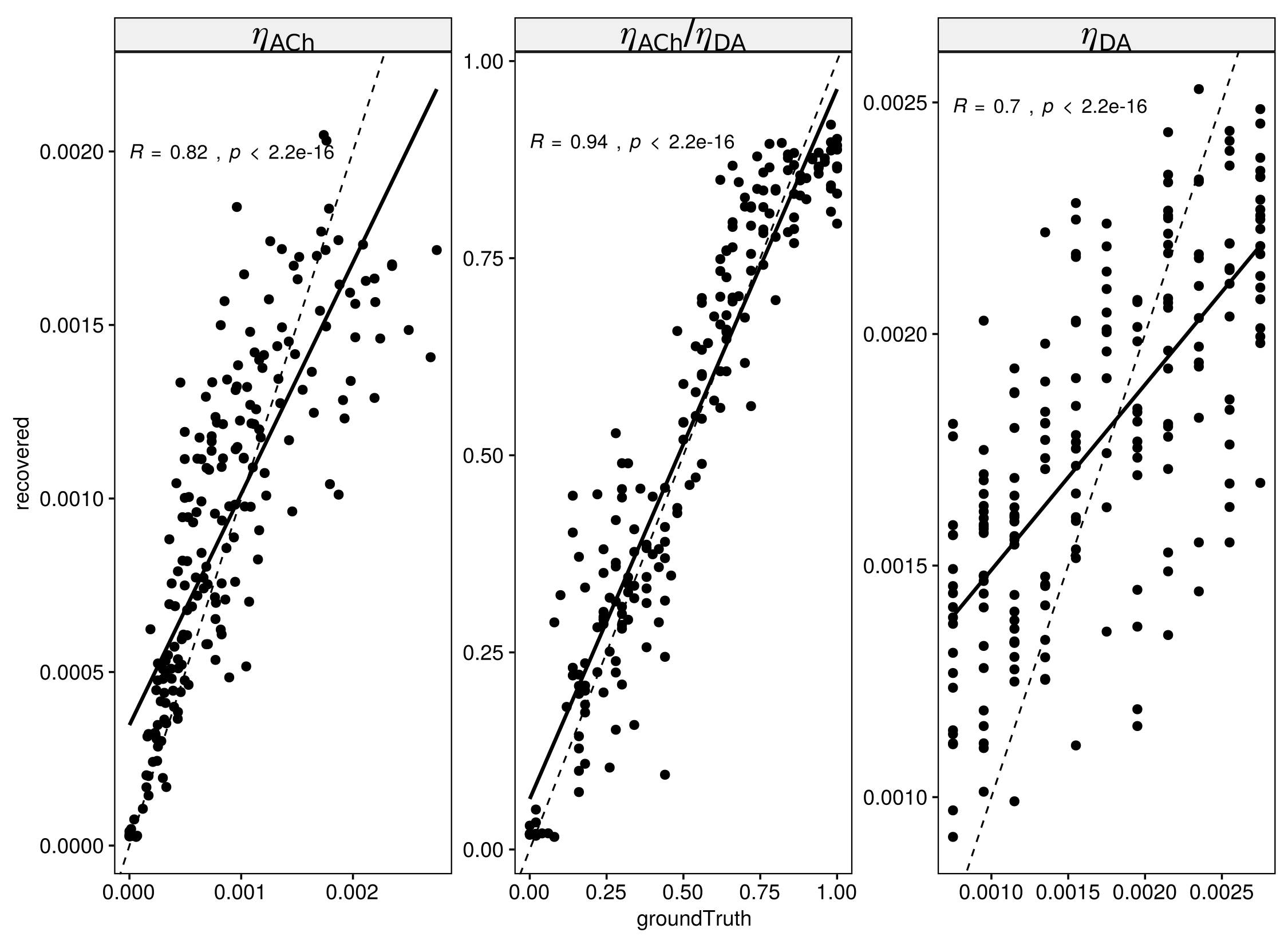

Supplement: S9 Fig — To establish parameter identifiability, we fit the model to 200 agents simulated from randomly-drawn parameter sets in the grid search. The estimated parameters are plotted against the values of the true parameters. Dotted line is the line of unity. (TIF) [file pcbi.1009017.s009.tif]

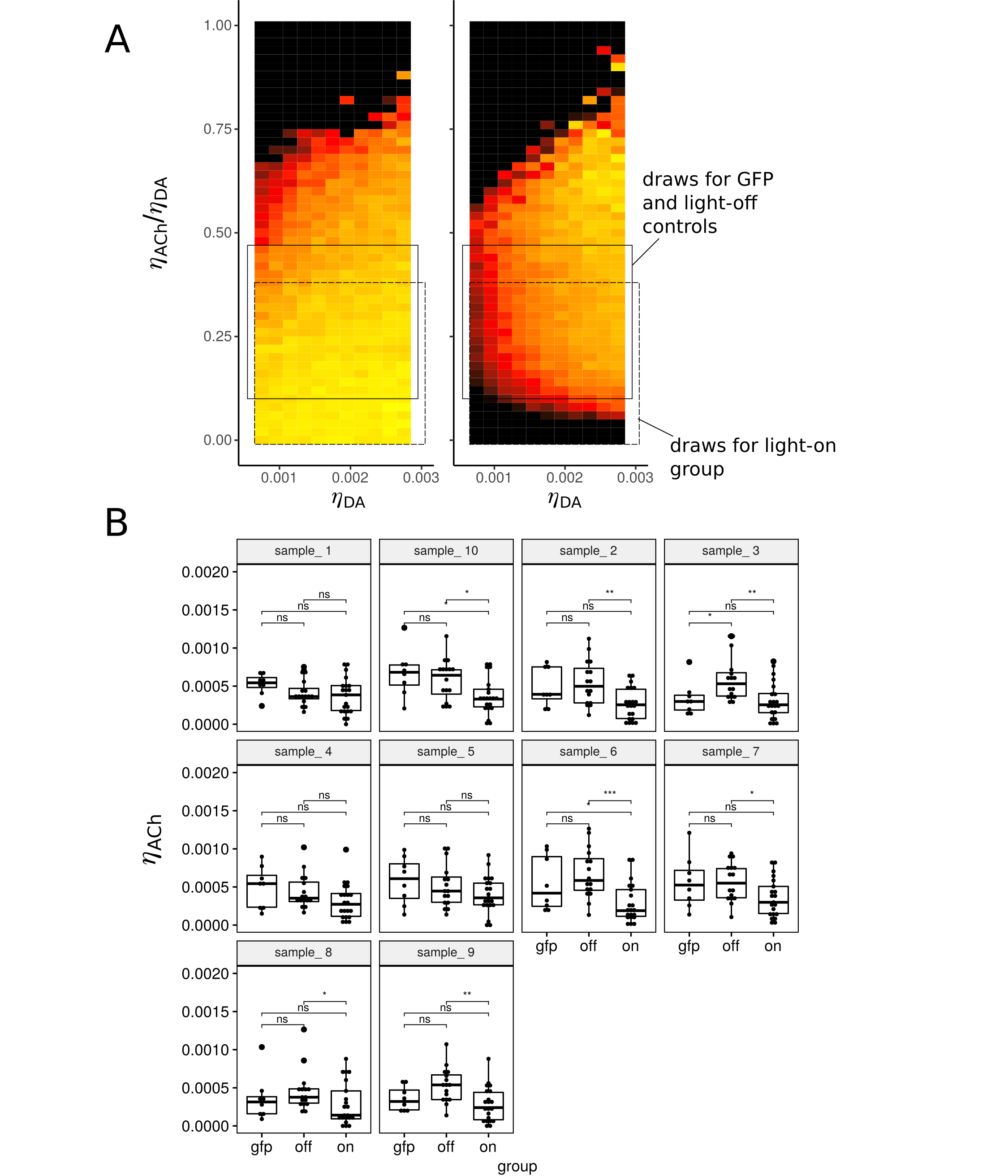

Supplement: S10 Fig — (A) Sets of parameters for the number of mice in the control groups (GFP, 8; light-off, 16) were drawn from the parameter space bordered in the solid black outline. 21 sets for light-on mice were drawn from an area (dashed outline) with reduced acetylcholine. (B) Group differences in parameter values were tested using the Kruskal-wallis test. Shown here are the results for 10 samples. (TIF) [file pcbi.1009017.s010.tif]

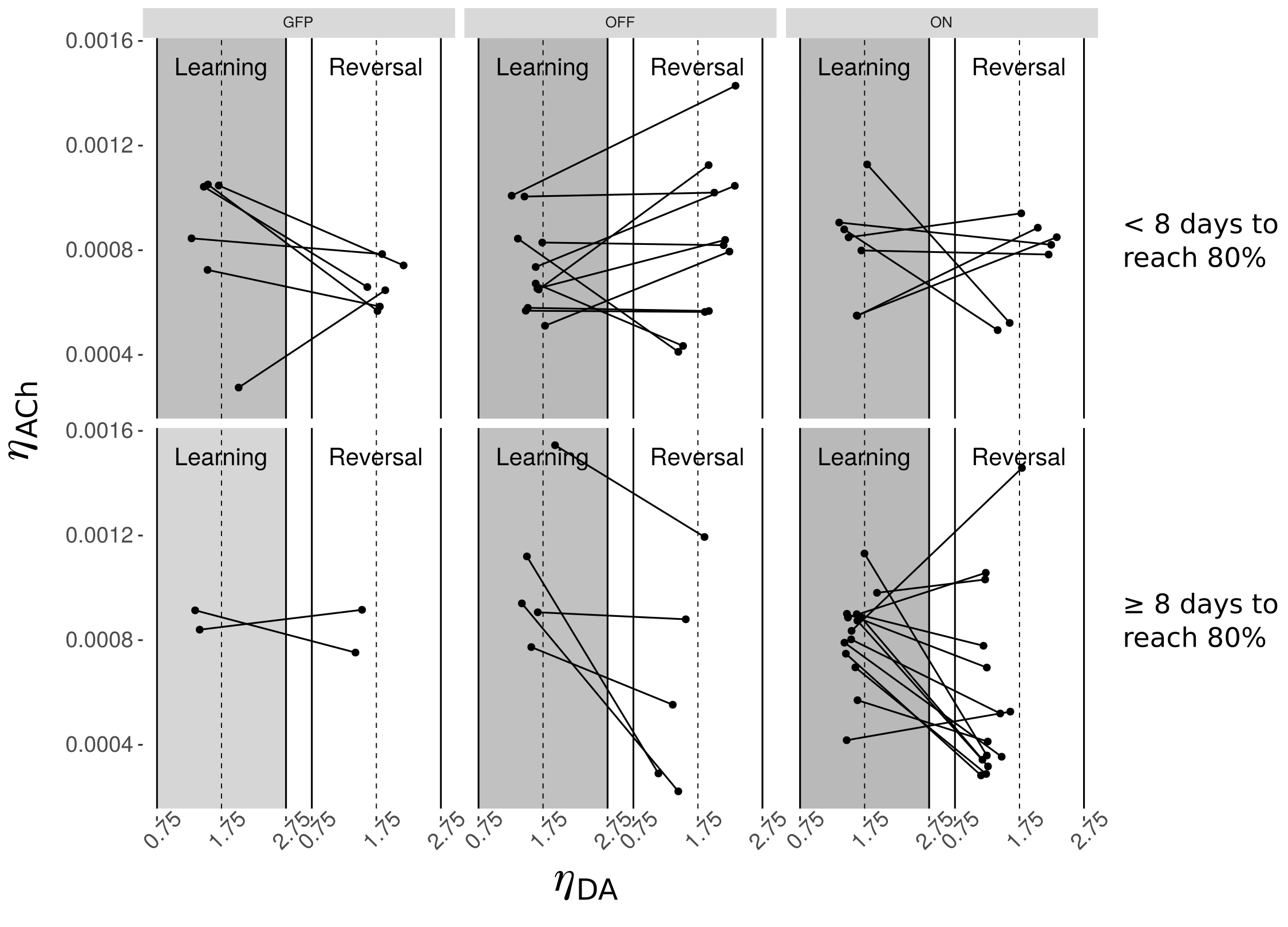

Supplement: S11 Fig — Here the model was fitted separately to data in each task stage, to see how well acetylcholine and dopamine values correlate across initial (points in grey area) and reversal learning. For most slow reversers (bottom panels), there appears to be a reduction in acetylcholine across initial and reversal learning. However, three light-on mice which did not show a strong preference for the old reward location on the first day of the reversal had high estimated ηACh. These trends are also shown matched to individual mice in the inset panels of S5–S7 Figs. (TIF) [file pcbi.1009017.s011.tif]

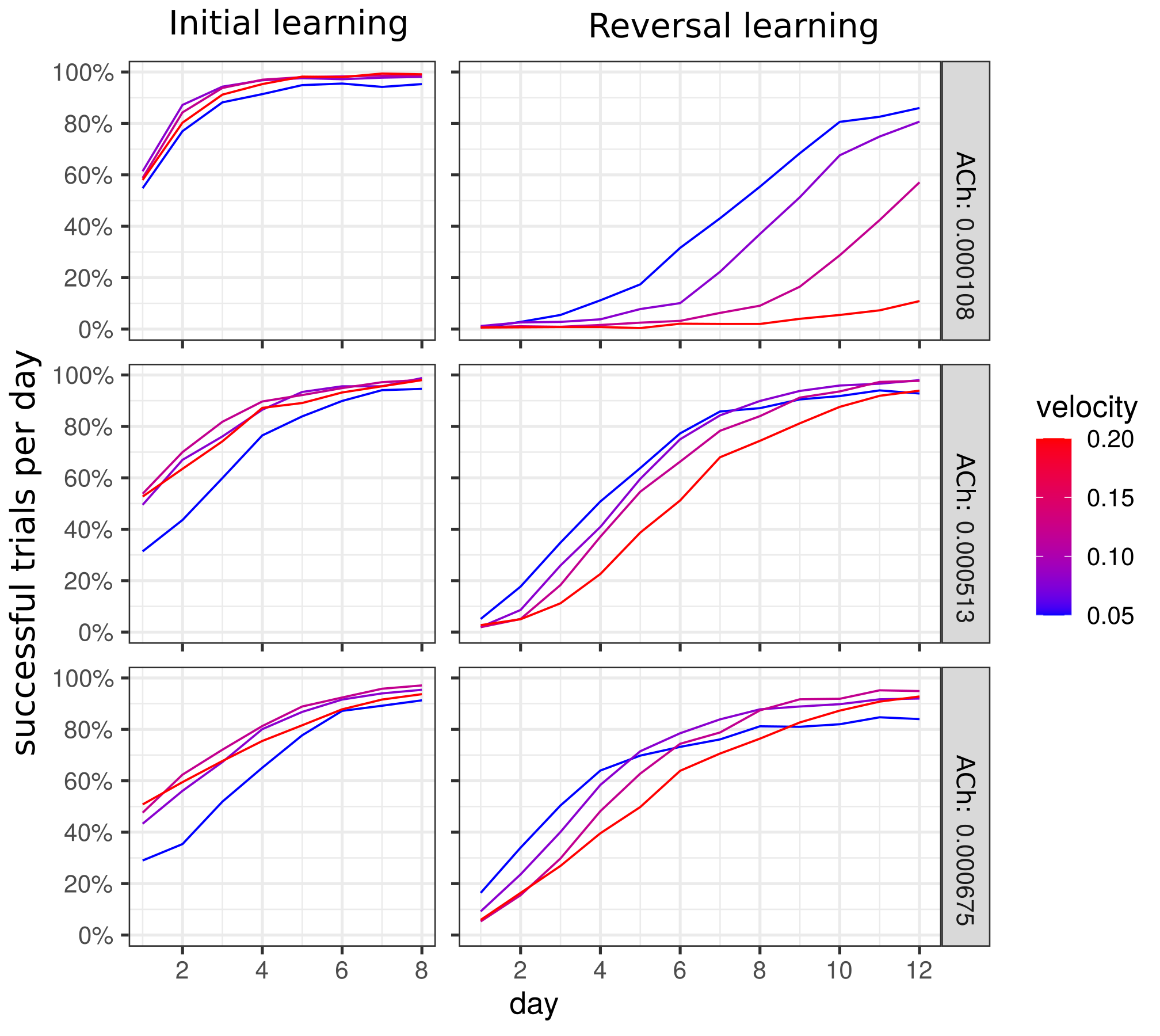

Supplement: S12 Fig — The effect of increasing agent speed on initial learning and reversal learning, at different acetylcholine levels, when ηDA = 0.00135. (TIF) [file pcbi.1009017.s012.tif]
